# Energy-dispersive Laue diffraction analysis of the influence of statherin and histatin on the crystallographic texture during human dental enamel demineralization

**DOI:** 10.1107/S1600576724007180

**Published:** 2024-09-25

**Authors:** Charbel Sakr, Mohammed Al-Mosawi, Tilman A. Grünewald, Philip Cook, Pieter Tack, Laszlo Vincze, Jean-Sebastien Micha, Paul Anderson, Maisoon Al-Jawad, Helga C. Lichtenegger

**Affiliations:** a University of Natural Resources and Life Sciences (BOKU) Vienna Austria; b European Synchrotron Radiation Facility Grenoble France; c University of Leeds Leeds United Kingdom; d Aix Marseille Univ. CNRS, Centrale Med, Institut Fresnel Marseille France; e Danish Technological Institute Høje Taastrup Denmark; f Ghent University Ghent Belgium; g Univ. Grenoble Alpes, UMR SyMMES, CEA-Grenoble/IRIG Grenoble France; h Queen Mary University of London London United Kingdom; Montanuniversität Leoben, Austria

**Keywords:** dental enamel, texture, Laue diffraction, crystallographic texture, hydroxyapatite, salivary proteins

## Abstract

Energy-dispersive Laue diffraction (EDLD) is a powerful method to obtain position-resolved texture information in inhomogeneous biological samples without the need for sample rotation. This study employs EDLD texture scanning to investigate the impact of two salivary peptides, statherin (STN) and histatin-1 (HTN) 21 *N*-terminal peptides (STN21 and HTN21), on the crystallographic structure of dental enamel. These proteins are known to play crucial roles in dental caries progression. Three healthy incisors were randomly assigned to three groups: artificially demineralized, demineralized after HTN21 peptide pre-treatment and demineralized after STN21 peptide pre-treatment. To understand the micro-scale structure of the enamel, each specimen was scanned from the enamel surface to a depth of 250 µm using microbeam EDLD. Via the use of a white beam and a pixelated detector, where each pixel functions as a spectrometer, pole figures were obtained in a single exposure at each measurement point. The results revealed distinct orientations of hydroxyapatite crystallites and notable texture variation in the peptide-treated demineralized samples compared with the demineralized control. Specifically, the peptide-treated demineralized samples exhibited up to three orientation populations, in contrast to the demineralized control which displayed only a single orientation population. The texture index of the demineralized control (2.00 ± 0.21) was found to be lower than that of either the STN21 (2.32 ± 0.20) or the HTN21 (2.90 ± 0.46) treated samples. Hence, texture scanning with EDLD gives new insights into dental enamel crystallite orientation and links the present understanding of enamel demineralization to the underlying crystalline texture. For the first time, the feasibility of EDLD texture measurements for quantitative texture evaluation in demineralized dental enamel samples is demonstrated.

## Abstract 2

Energy-dispersive Laue diffraction gives insights on local crystallographic texture variations in demineralized dental enamel and the role of salivary proteins in protecting the enamel structure.

## 1. Introduction

Biomineralized tissues such as bone and teeth are characterized by an exquisite arrangement of mineral crystallites that is tightly linked to their material properties (Weiner, 2008 ▸; Mann, 2001 ▸). In particular, the local crystallographic orientation of the mineral crystallites (crystallographic texture) is strictly controlled and can give valuable insight into mineralization and demineralization processes.

**Figure 1:**
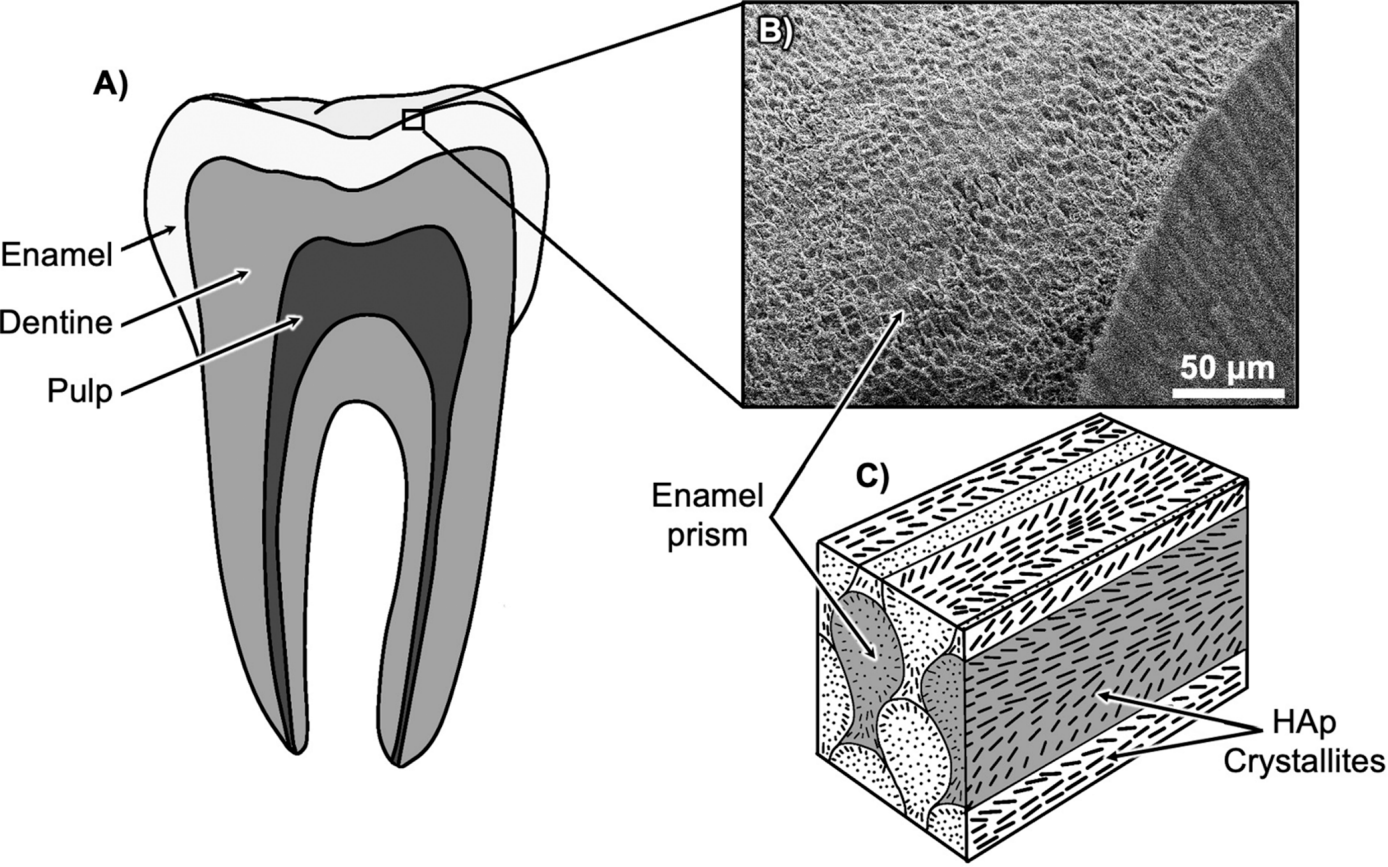
(*A*) A simplified diagram of a human tooth, illustrating its different components. (*B*) A scanning electron micrograph presenting the organizational pattern of enamel prisms. (*C*) A schematic representation detailing the 3D arrangement of crystallites within enamel prisms, redrawn from Habelitz *et al.* (2001 ▸) with permission from Elsevier.

Dental enamel, which forms the outermost layer of a tooth [Fig. 1 ▸(*A*)], consists of 95–98 wt% mineral content in the form of impure hy­droxy­apatite (HAp) (Elliott, 1997 ▸; Elliott *et al.*, 1998 ▸) as thin needle-like crystallites measuring approximately 28 nm in width, 68 nm in thickness and up to several micrometres in length (Kerebel *et al.*, 1979 ▸). The majority of these crystallites exhibit a hexagonal appearance with a high aspect ratio. They are tightly organized into bundles known as prisms, measuring 4–7 µm in diameter and extending from the enamel–dentine junction to the enamel surface (Simmer *et al.*, 2012 ▸; Shore *et al.*, 1995*b* ▸). In human enamel, the cross-sectional appearance of most enamel prisms resembles a keyhole shape, with head and tail regions (Shore *et al.*, 1995*b* ▸; Boyde, 1964 ▸). Crystallites in the head regions align along the long axis of the prism, while those at the tail are angled at about 45–70° to the direction of the prism long axes [Fig. 1 ▸(*C*)] (Shore *et al.*, 1995*a* ▸,*b* ▸; Robinson *et al.*, 1995 ▸; Poole & Brooks, 1961 ▸). The prisms are observed to be oriented approximately perpendicular to the enamel surface, enhancing the packing efficiency, flexibility, strength and wear resistance of enamel (Boyde, 1997 ▸). Towards the inner enamel, groups of prisms cross over or decussate in a stepwise manner, resulting in prism decussation and adding to the overall strength and resilience of the enamel (Boyde, 1997 ▸; Koenigswald *et al.*, 1987 ▸).

The current knowledge of the crystallographic texture of dental enamel is primarily based on X-ray diffraction (XRD) (Besnard *et al.*, 2023 ▸; Free *et al.*, 2022 ▸) and electron techniques (Beniash *et al.*, 2019 ▸; Koblischka-Veneva *et al.*, 2019 ▸). Most commonly, texture is determined using angular-dispersive XRD and sample rotation to obtain 3D crystallographic orientation information, as described in many textbooks [*e.g.* Cullity (1978 ▸)]. Sample rotation, however, is not feasible for scanning inhomogeneous biological samples larger than the beam size, because each rotation step results in a different irradiated volume, thus smearing the results. Various approaches have been used in the past to achieve spatially resolved texture scanning. Scanning XRD with area detectors (2D XRD) and no rotation has been applied to obtain limited texture information, making use of assumptions about the underlying texture. This approach can be suitable if the material is well studied, and as long as no structural changes due to disease, demineralization or other perturbations are expected. For more complex cases, 2D information is not sufficient. Other authors have also rotated sample slices, thus allowing parallax. This option was successfully employed for samples such as single bone trabeculae (Jaschouz *et al.*, 2003 ▸) or the scanning of a single bone osteon (layered structure) (Wagermaier *et al.*, 2007 ▸). Nevertheless, in the general case, information will be lost using this method.

Energy-dispersive Laue diffraction (EDLD) has been shown to give enhanced crystallographic information, since it can provide 3D crystallographic data in one exposure (without sample rotation) (Grünewald *et al.*, 2016 ▸). By using a white beam, diffraction signals from differently oriented crystallographic planes are obtained simultaneously (Laue diffraction). While conventional Laue diffraction has been used successfully on biomineralized tissues such as seashells (Tamura & Gilbert, 2013 ▸), it only works when the individual grain orientations can be separated in the pattern, thus limiting the degree of polycrystallinity. As a major difference, in EDLD an energy-dispersive pixelated area detector (where every pixel functions as a spectrometer) allows unambiguous identification of the contributing diffracting planes, even in strongly polycrystalline and nanocrystalline materials (Grünewald *et al.*, 2016 ▸). EDLD has been used, for example, for XRD of twinned lysozyme crystals (Send *et al.*, 2012 ▸), strain measurements in a Cu single crystal (Abboud *et al.*, 2014 ▸, 2017 ▸) or for mapping individual grains in steel (Abboud *et al.*, 2021 ▸).

**Figure 2:**
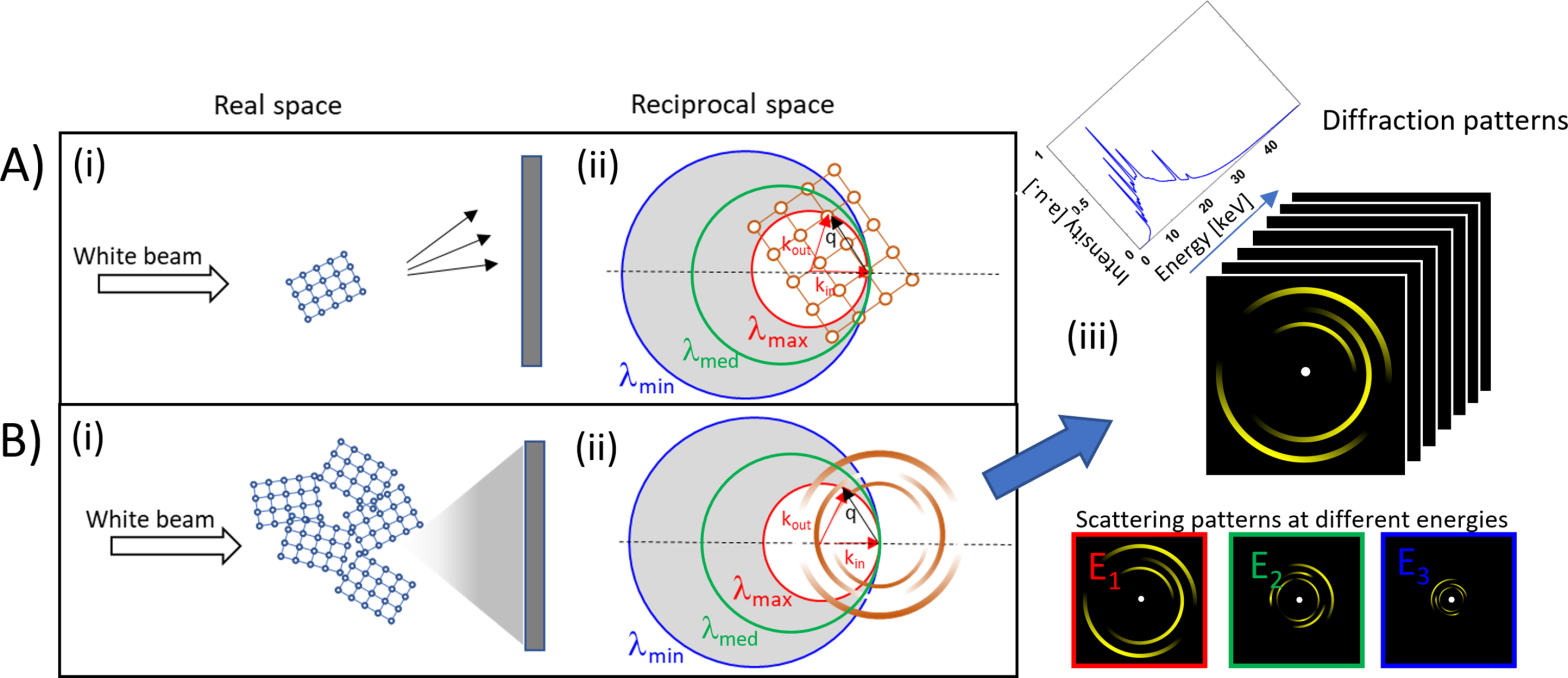
The concept of EDLD texture measurement. (*A*) Laue diffraction from a single crystal. (*A*)(i) The white beam is diffracted and single reflections hit the detector. (*A*)(ii) A representation in reciprocal space. Reflections occur where the Ewald sphere intersects the reciprocal crystal lattice. The Ewald sphere radius depends on the wavelength λ. In the white beam, a continuous spectrum from λ_min_ to λ_max_ is present. (*B*) Laue diffraction from a strongly polycrystalline sample. (*B*)(i) In this case, many orientations contribute, yielding a multitude of reflections. (*B*)(ii) A representation in reciprocal space. The reciprocal lattice is smeared out due to the many crystallite orientations. In the case of a completely random orientation, continuous hollow spheres would be present. For a given energy (or Ewald sphere) we can see a powder pattern (made of rings) on the detector. In the case of a texture, the rings show modulated intensity. Ewald spheres of different radii (originating from different incident wavelengths) intersect the reciprocal object at different points, thus providing information about intensity variations in the rings (spheres). (*B*)(iii) The diffraction patterns recorded at different energies in one shot show varying signals at different energies, which can be used to obtain information about the 3D orientation distribution of the crystallites (crystallographic texture).

In Fig. 2 ▸, a geometric representation of EDLD is shown. When a crystalline sample is irradiated with an X-ray beam, a diffraction signal will be recorded [shown schematically in Fig. 2 ▸(*A*) for a single crystal and Fig. 2 ▸(*B*) for a polycrystalline material]. According to diffraction theory, the image on the detector corresponds to an intersection of the reciprocal crystallographic lattice (the Fourier transform of the lattice in real space) with the Ewald sphere of radius *k*, where *k* = 2π/λ and λ is the wavelength of the incident beam. As a consequence, in the case of a polychromatic beam the diffracted pattern can be described as a set of sections of Ewald spheres with different radii through reciprocal space [Figs. 2 ▸(*A*)(ii) and 2 ▸(*B*)(ii)]. Thus 3D information in reciprocal space can be obtained from the different curvatures of the Ewald spheres. In this way, the energy resolution becomes the third dimension in space. For additional information about this method, the reader is also referred to the article by Grünewald *et al.* (2016 ▸), where the principle of EDLD texture measurements was demonstrated qualitatively on carbon fibres and lobster cuticle. In a related approach, Park and co-workers have employed energy-dispersive diffraction with ten point detectors in an array to image the composition and texture in a shark centrum (Park *et al.*, 2022 ▸; Stock *et al.*, 2022 ▸). While they achieved tomography using a slit system, they were limited to much lower spatial resolution and concentrated on imaging rather than detailed texture analysis.

In the present paper we demonstrate, for the first time, the feasibility of EDLD texture measurements for quantitative texture evaluation in demineralized dental enamel samples.

Dental caries, commonly known as tooth decay, is a multifactorial bacterial biofilm-mediated non-communicable disease that results from dietary, behavioural, environmental and psycho-social factors. It is a dynamic disease that causes a net loss of minerals from dental hard tissues, leading to the formation of carious lesions (Machiulskiene *et al.*, 2020 ▸). HAp crystallites are reactive in acidic solutions. When the pH in the oral environment drops below a critical pH value (typically pH 5.5) (Anderson *et al.*, 2001 ▸), an ionic imbalance occurs which progressively leads to the loss of ionic species and the de­mineralization of enamel. Under normal physiological conditions, the salivary ion content and buffering capacity can counteract the demineralization process, with a delicate equilibrium where demineralization and remineralization (redeposition of mineral phases) occur. Changes in the oral microenvironment, such as significant carbohydrate intake, can impair this equilibrium and favour demineralization. When the microenvironment reaches pH values above 7.0 and there is an availability of calcium and phosphate ions, re­mineralization occurs, reversing the demineralization effect (Ionescu *et al.*, 2022 ▸).

Saliva contains a variety of electrolytic components (de Almeida *et al.*, 2008 ▸), including peptides like statherins (STNs) and histatins (HTNs) which are among the numerous proteins that play a role in a wide range of biological functions (Margolis & Moreno, 1992 ▸).

HTNs, a family of histidine-rich peptides, are small basic non-immunologic salivary proteins. One of their functions is their bactericidal and fungicidal effects, which occur through the binding of positively charged HTNs with biological membranes, leading to the disruption of their architecture and permeability. Additionally, HTNs have been found to participate in acquired film formation and inhibit histamine release, suggesting a potential role in oral inflammation (Kaufman & Lamster, 2002 ▸; de Almeida *et al.*, 2008 ▸; Dodds *et al.*, 2005 ▸). HTNs are capable of binding to HAp, which is thought to be facilitated by the negatively charged amino terminal region of HTNs. This binding interaction is postulated to play a role in preventing the precipitation of calcium phosphate from saturated saliva. Moreover, this interaction effectively inhibits crystal growth, leading to enhanced stability of HAp on the tooth surface (Dowd, 1999 ▸). Siqueira *et al.* (2010 ▸) found that intact HTN proteins bind to the enamel surface and provide protection against proteolytic degradation, indicating their potential effectiveness in protecting enamel from demineralization in acidic environments (Siqueira *et al.*, 2010 ▸).

STN is a tyrosine-rich non-immunologic acidic salivary protein with a molecular weight of 5.38 kDa (Kaufman & Lamster, 2002 ▸; de Almeida *et al.*, 2008 ▸; Dodds *et al.*, 2005 ▸; Dowd, 1999 ▸). The *N*-terminal region of the molecule contains negatively charged residues that serve as binding sites for HAp (Dowd, 1999 ▸). The binding of STN to HAp is characterized by a complex interaction (Chen *et al.*, 2008 ▸; Raghunathan *et al.*, 2006 ▸) between the α-helix of STN and the (001) plane of HAp (Makrodimitris *et al.*, 2007 ▸). Similarly to HTNs, STN prevents the formation of salivary and dental calculus by inhibiting the spontaneous precipitation of calcium phosphate salts and the growth of HAp crystallites on the tooth surface (Kaufman & Lamster, 2002 ▸; de Almeida *et al.*, 2008 ▸; Dodds *et al.*, 2005 ▸; Dowd, 1999 ▸), but to a higher extent than HTNs (Dowd, 1999 ▸). It is believed that the highly negatively charged amino-terminal segment of STN is likely to be the main inhibitory part of the peptide (Lenander-Lumikari & Loimaranta, 2000 ▸). By maintaining saliva in a supersaturated state with respect to calcium and phosphate salts, STN helps prevent enamel demineralization. STN adsorbed to HAp is also believed to bind to bacteria and selectively mediate bacterial adhesion to tooth surfaces (Kaufman & Lamster, 2002 ▸; de Almeida *et al.*, 2008 ▸; Dodds *et al.*, 2005 ▸; Dowd, 1999 ▸). Kosoric *et al.* (2007 ▸) studied the effect of synthetic truncated statherin-21 (STN21) on artificial carious and erosive lesions and found a 40% decrease in mineral loss over three weeks.

In the present study, we examine the impact of HTN and STN 21 *N*-terminal peptides (HTN21 and STN21) on the demineralization process and on the associated crystallographic texture of human dental enamel using energy-dispersive Laue diffraction (EDLD) texture measurements. Due to the one-shot nature of the employed method and the absence of sample rotation, we were able to perform small step scans across slices of tooth enamel with a micrometre-sized X-ray beam and study the local texture at every measurement point. Through quantitative evaluation, we gain insight into the orientation patterns in the differently treated samples. This insight could guide the development of targeted treatments for conditions affecting dental enamel. In essence, our study not only demonstrates the practical applications of EDLD but also helps to obtain a mechanistic understanding of the dynamic alteration of enamel demineralization induced by peptide treatments.

## 2. Methods

### 2.1. Peptide preparation

A 10 mmol l^−1^ phosphate-buffered saline solution was prepared by dissolving phosphate-buffered saline powder (Sigma–Aldrich, UK) in deionized water. The final solution had a pH of 7.4 and contained 0.138 mol l^−1^ NaCl and 0.0027 mol l^−1^ KCl.

Truncated 21 *N*-terminal STN21 and HTN21 peptides were synthesized by fluorenylmethoxycarbonyl protecting group solid-phase peptide synthesis (Fmoc SPPS) and purified (98% pure) by high-performance liquid chromatography as tri­fluoro­acetate salts (Peptide Protein Research Ltd, UK). The peptides were measured using a UMX2 ultra-microbalance (Mettler Toledo, Richmond Scientific Limited, Lancashire, UK). STN21 and HTN21 were then dissolved in phosphate-buffered saline (PBS) solution at a concentration of 200 nmol ml^−1^. The concentration chosen for this study was within the range of STN concentrations used by Kosoric *et al.* (2007 ▸) to investigate the impact on HAp demineralization of a concentration up to ten times higher than that found in saliva.

### 2.2. Sample selection and preparation

A total of three healthy incisors were collected with informed consent from patients treated at Barts and the London Dental Hospital (London, UK) clinics (QMREC 2011/99). They were randomly separated into three groups: DC – artificially demineralized, DH – demineralized with trun­cated histatin (HTN21) and DS – demineralized with STN21.

The demineralization solution was prepared by diluting a stock solution of 100 mmol l^−1^ acetic acid (AnalaR VWR) with deionized water and adjusting the pH to 4.0 using a 1000 mmol l^−1^ NaOH solution (Sigma–Aldrich, UK).

Sample DC was immersed in a demineralization solution of pH 4.0, consisting of 100 mmol l^−1^ acetic acid, for 2 d. Following this, it was rinsed with distilled water and transferred to a PBS solution for 24 h. After the PBS treatment it was immersed once again in the demineralization solution for an additional 2 d. Samples DH and DS underwent similar treatment to sample DC. However, in the case of sample DH, it was transferred to a PBS solution containing HTN21 at a concentration of 200 nmol ml^−1^, while sample DS was transferred to a PBS solution containing STN21 at a concentration of 200 nmol ml^−1^. All steps were carried out at 25.0 ± 2.0 ˚C.

The specimens were embedded in fast-curing acrylic cold-mounting resin (ClaroCit Kit, Struers, Ballerup, Denmark). Each of the embedded teeth was cut through the mid-point perpendicular to the bucco-lingual surface using a Struers Accutom-5 diamond saw (Struers, Ballerup, Denmark) to produce a 300 µm thick mid-slice for each tooth. The slices were then wet-polished down to 20 µm using 800 grit silicon carbide abrasive paper (Wetordry Tri-M-Ite paper, 3M, Minnesota, USA), as described in more detail in the supporting information (Note S1)

### 2.3. Energy-dispersive Laue diffraction

EDLD measurements were carried out on the CRG-InterFace beamline BM32 at the European synchrotron (ESRF). A polychromatic beam with a continuous energy distribution from 5 to 23 keV was used. This beam was obtained through a two-step demagnification of the white beam coming from a bending magnet (0.85 T): Primary mirrors in the optics hutch were employed to first focus the beam vertically onto a secondary source downstream. Subsequently, Kirkpatrick–Baez (KB) mirrors located close to the sample further reduced the beam size to 1 µm as a compromise regarding the grain size and the number of illuminated grains. To minimize air scattering, a helium-purged lead collimator was placed between the exit window of the KB mirror and the sample.

The diffraction signal was recorded by an energy-dispersive pixelated detector of pnCCD type (SLcam). The active area consisted of 264 × 264 pixels, each with a pixel size of 48 × 48 µm. Each pixel could record an energy range from 1.7 to 40 keV using 1024 channels. The chip was read out at a frequency of 400 Hz, resulting in a maximum count rate of approximately 600 000 counts per second (cps) for the entire chip, or approximately 10 cps for an individual pixel. Due to the small size of the active area, the detector was mounted on a home-built manually adjustable goniometer stage. While the sample remained stationary, the stage allowed moving the detector (SLcam) around the sample in a 2 × 2 array to detect diffraction at larger angles, minimizing geometric distortion of the diffraction images.

The primary beam was blocked by a small gold beamstop (200 µm diameter, 4 mm length) mounted on a spherical Kapton shell with a thickness of 100 µm to allow the scattered radiation to pass through. Both the beamstop and the sample were mounted on downstream extensions, allowing a small sample-to-detector distance and, simultaneously, rotation of the SLcam around the centre of rotation of the home-built stage to access larger scattering angles. The active area of the detector was placed 22 mm behind the sample. A range of up to 2θ = 40° was covered by four slightly overlapping detector positions.

**Figure 3:**
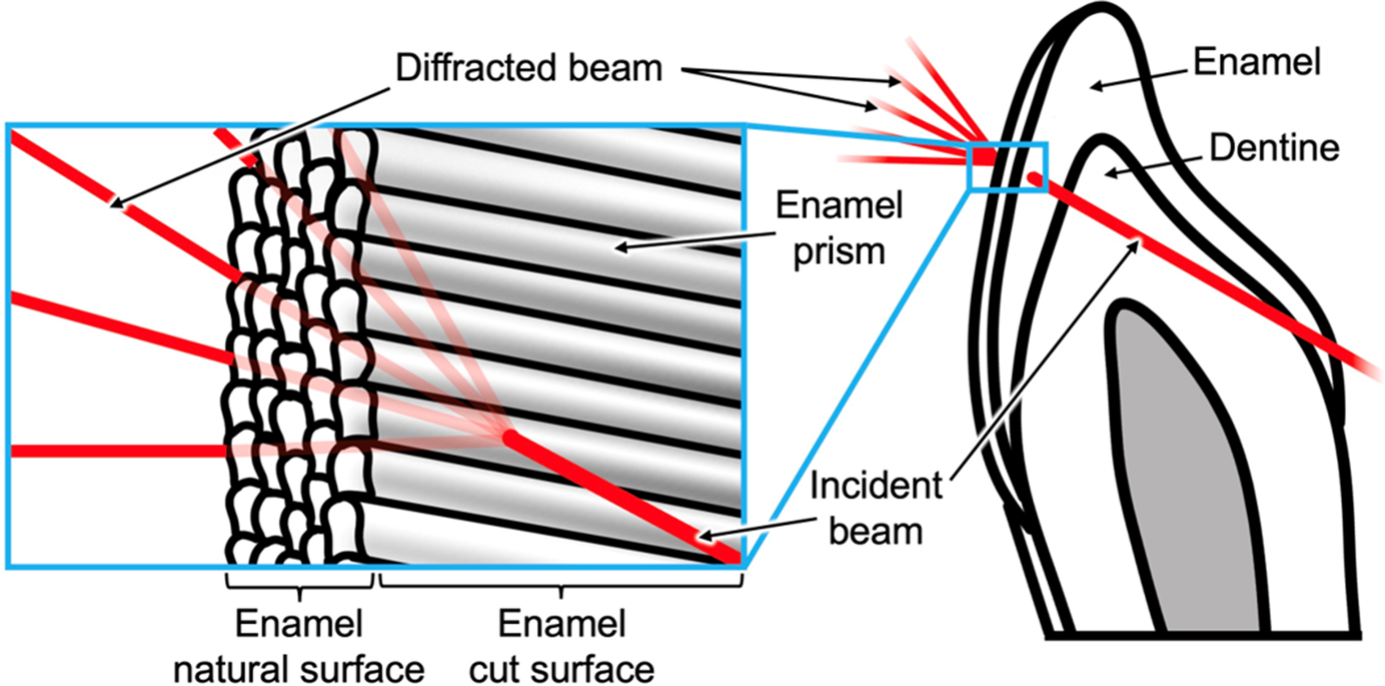
A schematic diagram illustrating a tooth slice and the diffraction geometry in the synchrotron experiment, with a close-up showing the beam direction in relation to the orientation of the enamel prisms.

The sample was affixed to a silicon nitride membrane and placed on a sample holder which was mounted on an automated *x*-*y*-*z* stage and brought into the centre of the home-built goniometer stage. Line scans with a step width of 5–7 µm were performed on the sample, starting at the enamel surface and extending 250 µm into the enamel. The sample slice was always kept perpendicular to the beam and was not rotated. Fig. 3 ▸ shows the measurement geometry in relation to the sample and the enamel prisms located in the tooth section.

### 2.4. Data processing

Data integration was performed using Python routines based on *pyFAI* (Kieffer *et al.*, 2020 ▸) and complemented with home-written routines. The calibration of the geometry was carried out without external calibrant, but used the diffraction patterns of each measured enamel sample and the known *d* spacing of HAp. Due to the slight inaccuracies in the detector position on the manually movable goniometer stage, this calibration was done for every sample and every detector position separately. As this corresponds to intrinsic calibration, the *d*-spacing information was not further evaluated, but evaluation was focused on crystallographic texture only.

The data were normalized to account for variations in incident intensity, detector sensitivity and sample absorption at different energies and were background corrected before further evaluation, as described in Note S2 in the supporting information.

**Figure 4:**
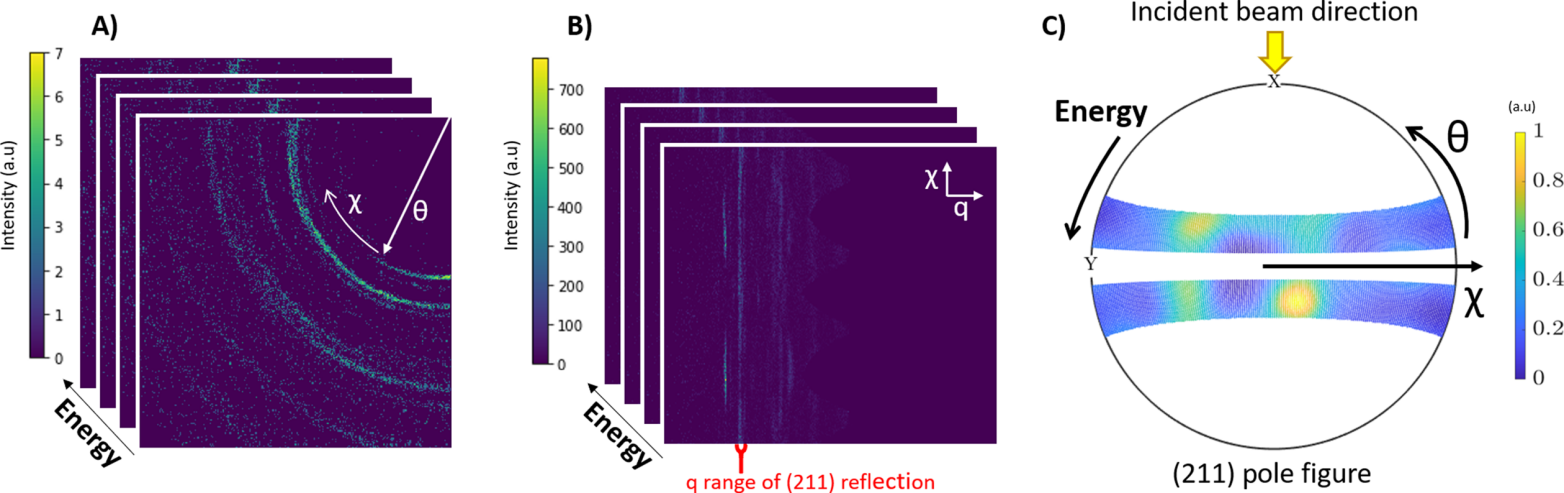
(*A*) A diffraction image stack (data cube containing 2D diffraction images at different energies) obtained from one position of the detector. (*B*) A (*q*, χ, *E*) data cube obtained by integration (caking) from diffraction images of all detector positions. Bragg reflections appear as vertical lines of varying intensity. For each reflection to be evaluated, a narrow *q* range around the reflection was chosen and integrated over *q*. The resulting χ–*E* maps for each reflection can be directly transformed into χ–θ maps and displayed as (*C*) a pole figure for an individual reflection.

The four individual slightly overlapping energy-dispersive diffraction image stacks resulting from the four detector positions [Fig. 4 ▸(*A*)] were first integrated separately using *PyFAI*. In this step, the diffraction images at individual energies were integrated and converted to intensity maps versus the scattering vector magnitude *q*, with
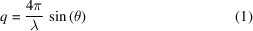
(where θ is half the scattering angle and λ is the wavelength of the incident radiation), and the azimuthal angle χ, thus yielding a new (*q*, χ, *E*) data cube with the energy as the third dimension [Fig. 4 ▸(*B*)]. The four new individual data cubes from the four detector positions were merged into one data cube. Each detector position covered an azimuthal range of slightly more than 90°, thus creating an overlap. During merging, each cube was cut to 90° exactly and the overlap was omitted. The final merged data cube covers the full 360° azimuthal range. Bragg reflections from three crystallographic planes (002), (211) and (130) were chosen for texture evaluation.

For further processing, the intensity in the (*q*, χ, *E*) data cube was integrated in a small *q* range around the respective reflection, thus yielding (χ, *E*) maps of intensity. Since at a given *q* the energy *E* is directly associated with the scattering angle 2θ by equation (1) and by *E* = *hc*/λ, we get (χ, θ) maps of intensity, which are readily translated into pole figures [Fig. 4 ▸(*C*)]. Note that pole figures have to be calculated for each reflection separately. The above procedure was performed for each data point in the scan lines across the enamel surface.

Only part of the pole figure can be covered in this way [Fig. 4 ▸(*C*)]. This because of the finite energy range accessible in the experiment and the finite range of scattering angles 2θ due to geometric restrictions. In our experiment, the 2θ range was well adapted to the accessible energy range of 5–23 keV.

### 2.5. Texture evaluation

Experimental pole figures (PFs) of the selected reflections from (002), (211) and (310) were imported into the MATLAB-based software *MTEX* (Hielscher & Schaeben, 2008 ▸). After setting the crystallographic parameters of HAp (*i.e.* space group, lattice parameters) and the crystal coordinate system according to the sample coordinate system (or reference coordinate system), *MTEX* can be used to give a direct estimation of the orientation distribution function (ODF) *f*(*g*) defined by the three Euler angles (φ_1_, Φ, φ_2_) for each measurement point (Cho *et al.*, 2004 ▸):
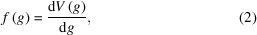
with d*g* = sinΦ dφ_1_ dΦ dφ_2_/8π^2^, and d*V*(*g*) being the volume portion of crystallites having orientations within an infinitesimal orientation element d*g*. The ODF is a representation of the full crystallographic texture and can be read as a probability map of 3D crystallographic orientations of all crystallites in each scan point. Furthermore, the ODF can be used to reconstruct pole figures. *MTEX* uses the series expansion method involving spherical harmonics to estimate ODFs (Hielscher & Schaeben, 2008 ▸).

**Figure 5:**
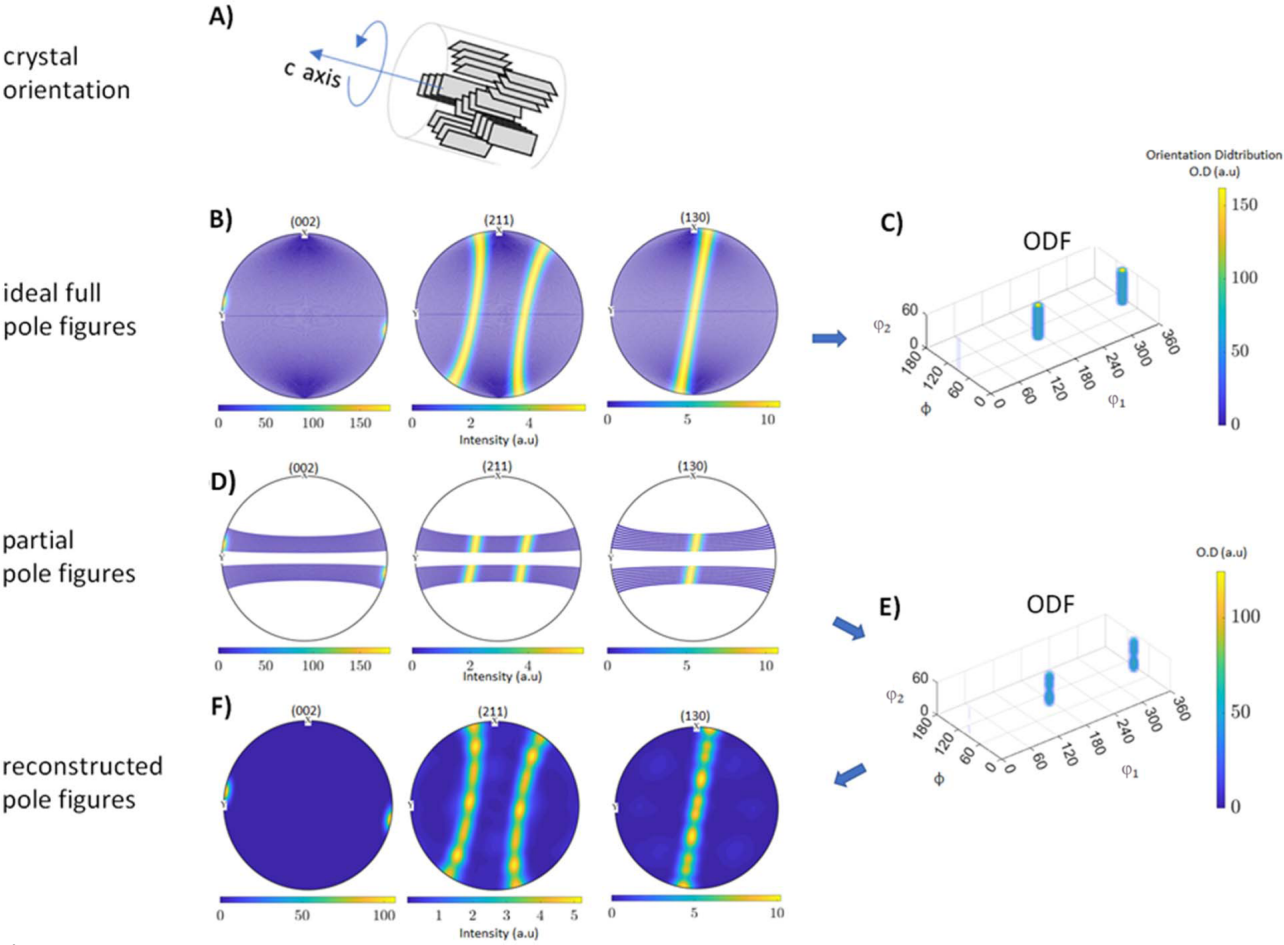
(*A*) The arrangement of HAp crystallites in a fibre texture about the crystallographic *c* axis. (*B*) Simulated full PFs, (*C*) the estimated ODF from the full PFs, (*D*) incomplete PFs in the experimentally accessible range, (*E*) the estimated ODF from the incomplete PFs and (*F*) reconstructed pole figures from the ODF obtained from the partial pole figures.

In the following we show a simulation to exemplify the procedure. In the ideal case, pole figures would be complete with full coverage of reciprocal space. In practice this is not possible, since measured pole figures consist of single measurement points or limited areas, depending on the chosen setup. In our case, in the absence of sample rotation, the coverage is limited by the experimentally available energy range and the range of scattering angles 2θ [see also Fig. 4 ▸(*C*)]. In Fig. 5 ▸(*A*) we show the consequence of this limitation in the case of a fibre texture about the crystallographic *c* axis. A fibre texture about the *c* axis, sometimes somewhat imperfect, is a typical crystallographic orientation of HAp crystallites found in long bones (Almer & Stock, 2005 ▸; Wagermaier *et al.*, 2007 ▸; Jaschouz *et al.*, 2003 ▸; Wenk & Heidelbach, 1999 ▸; Grünewald *et al.*, 2020 ▸; Reznikov *et al.*, 2018 ▸) and tooth enamel (Al-Jawad *et al.*, 2007 ▸). It means a parallel arrangement of crystallites along their *c* axis but random rotation about it [Fig. 5 ▸(*A*)]. Ideal pole figures for (002), (211) and (310) are shown in Fig. 5 ▸(*B*) and the corresponding ODF in Fig. 5 ▸(*C*). The ODF shows two columns in the vertical φ_2_ direction, separated by 180°, which is representative of a fibre texture about the *c* axis. The position of the columns in the φ_1_–Φ plane of the ODF gives information about the tilt of the *c* axis.

Incomplete PFs, simulated in the experimentally accessible range, are shown in Fig. 5 ▸(*D*). The corresponding ODF looks very similar to the one obtained from ideal pole figures, but with a slight granularity in the columns [Fig. 5 ▸(*E*)]. From the ODF, complete pole figures were reconstructed [Fig. 5 ▸(*F*)]. Note that the overall characteristic of a fibre texture is still preserved, although here a granularity and inhomogeneity of intensities are also observed in the stripes in the (211) and (130) pole figures, respectively. These reconstruction artefacts are due to the series expansion method involving spherical harmonics used by *MTEX*. When working with incomplete data in the pole figures and performing the transformation of these data into Fourier space, the missing information can cause aliasing, leading to periodic variations in intensity.

Experimental pole figures of enamel were used to calculate ODFs at every scan point. Fibre orientations were fitted using the implemented *MTEX* function ‘fibrefit’, and the positions of the fibres in the reference coordinate system are given in Cartesian coordinates as an output. These coordinates are later used to display the orientation of the fibres in the reference coordinate system and to obtain the tilt angle of the fibres with the enamel surface and the misorientation angle between fibres. In the case of several fibre populations present in the sample, the ‘fibrefit’ algorithm was used to determine the most dominant fibre component. Once these results were obtained, the columns in the ODF representing this first component were erased from the ODF and the ‘fibrefit’ algorithm was used to find the next dominant component. This procedure was repeated until only noise was fitted.

ODFs were also used to obtain quantitative information. The relative volume of crystals close to a certain orientation is given by
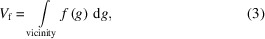
with d*g* = sinΦ dφ_1_ dΦ dφ_2_/8π^2^ and the integral being computed only over those regions of Euler space whose orientation distance ω from the ideal texture component of interest is less than ε angular units (Cortie, 1997 ▸). In practice, the volume fraction of crystallites *V*_f_ with an orientation in the vicinity of a given fibre orientation was determined using the implemented *MTEX* function ‘fibrevolume’ [this function takes as input the ODF *f*(*g*), the crystal directions, the Cartesian coordinates of the fibre orientation and the maximum orientation distance ε]. The ODF can also be used to determine the relative quantities of crystallites in a sample comprising different fibre orientations.

A useful parameter describing the degree of preferred orientation, or texture strength, is the texture index, given by
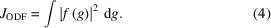
*J*_ODF_ = 1 for a completely random orientation and becomes infinite in the case of all crystallites having one single orientation (Mainprice *et al.*, 2015 ▸). It was determined using the *MTEX* function ‘textureindex’.

## 3. Results

### 3.1. Single and multiple crystallite orientation populations

**Figure 6:**
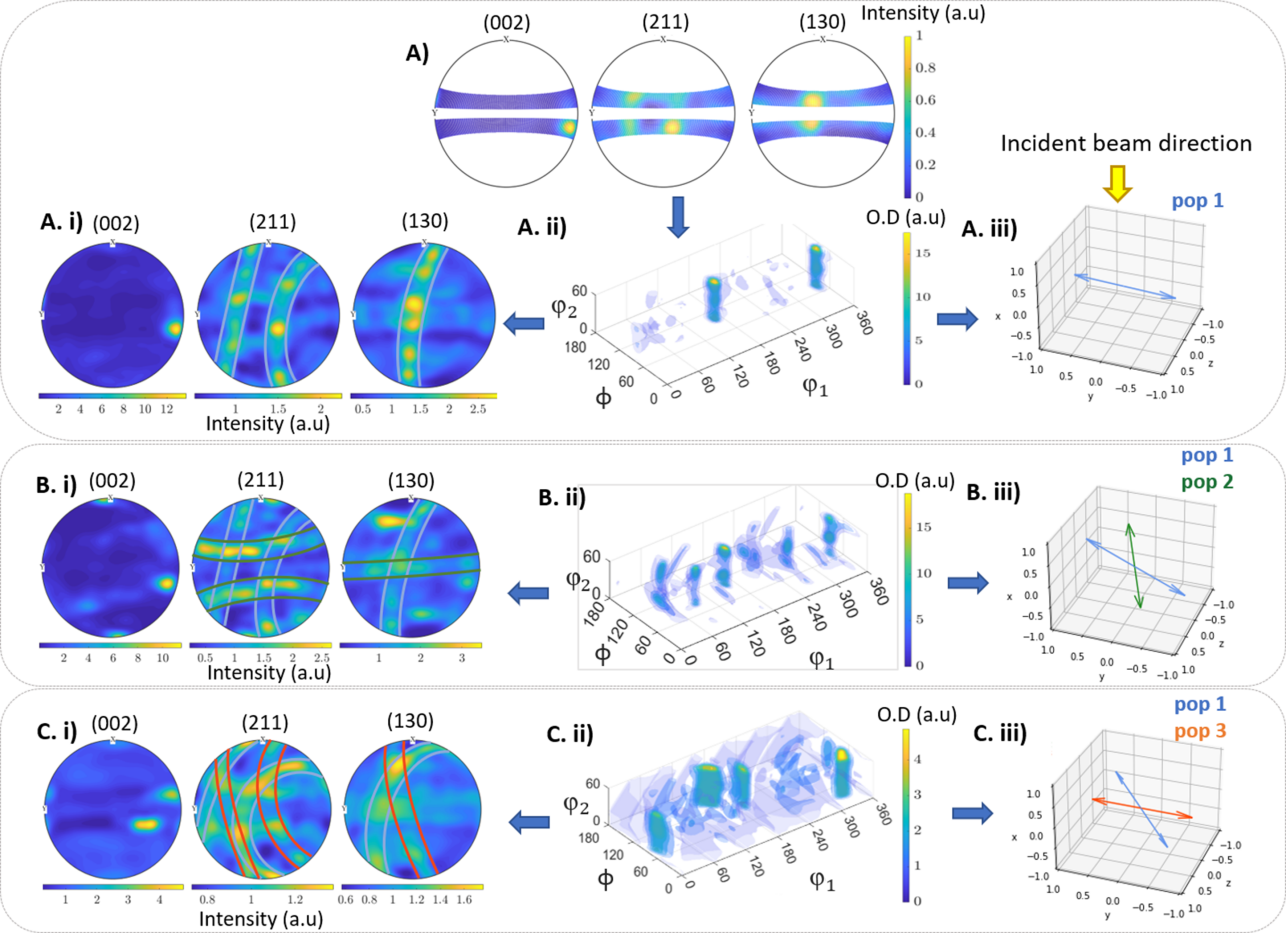
(*A*) Experimental pole figures. (*A*)(i) The estimated ODF and (*A*)(ii) reconstructed pole figures showing a single orientation population with an out-of-plane tilt of 7° on average with the enamel surface. (*A*)(iii) The fibre axis in the reference coordinate system. (*B*)(i) and (*B*)(ii) Reconstructed pole figures and ODF, respectively, showing two populations offset by 68°. (*B*)(iii) Both fibre axes in the reference coordinate system. (*C*)(i) and (*C*)(ii) Reconstructed pole figures and ODF, respectively, showing two populations offset by a value of 36°. (*C*)(iii) Both fibre axes in the reference coordinate system.

In Figs. 6 ▸(*A*), 6 ▸(*A*)(i), 6 ▸(*A*)(ii) and 6 ▸(*A*)(iii) we provide an experimental example showcasing a typical fibre texture around the *c* axis. The incomplete experimental pole figures obtained by EDLD [Fig. 6 ▸(*A*)] yield pole figure reconstructions of HAp close to those expected for an ideal fibre texture [Fig. 6 ▸(*A*)(i)] but showing a certain graininess in the (211) and (130) rings [an effect that was also demonstrated in Section 2.5 and Fig. 5 ▸(*F*)]. The 3D orientation of the crystallographic *c* axis in real space is shown in Fig. 6 ▸(*A*)(iii).

At several scan points in the sample, however, multiple crystallites orientations were observed. To illustrate this, we present examples of reconstructed pole figures in Figs. 6 ▸(*B*)(i) and 6 ▸(*C*)(i). In Figs. 6 ▸(*B*)(i), 6 ▸(*B*)(ii) and 6 ▸(*B*)(iii), a combination of two fibre textures (two populations) with different orientations are visible as two sets of spots in the (002) pole figure and two sets of rings in the (211) and (130) pole figure, respectively. The angular difference between the *c*-axis orientations of the populations is 68° [Fig. 6 ▸(*B*)(iii)]. In Figs. 6 ▸(*C*)(i), 6 ▸(*C*)(ii) and 6 ▸(*C*)(iii), two crystallite populations with an angular difference in the *c*-axis orientation of 36° are shown. In order to demonstrate further the robustness of the reconstruction, we show simulations of ideal fibre textures, oriented in the same way as in Figs. 5 ▸(*B*) and 5 ▸(*C*), in Figs. S1 and S2 (see the supporting information). Note that, in the experimentally derived ODFs, intensity is also found outside the vertical columns that represent the fibre orientations. This is indicative of texture imperfections, such as imperfect fibre texture and additional different crystallite orientations, which are likely to occur in biological materials.

Due to the wealth of 3D information contained in the measured PFs and the calculated ODFs, we can determine the actual 3D orientation of the crystallite populations with respect to each other. This capability stands in contrast to conventional 2D XRD with a monochromatic beam and an area detector, as 2D XRD techniques are unable to detect out-of-plane tilts directly.

### 3.2. Texture in demineralized and remineralized dental enamel

From the analysis of measured PFs and calculated ODFs, several key parameters regarding enamel crystallites were determined at each scan point. These parameters include:

(i) The crystallite orientation represented by the tilt angle of the *c* axis with respect to the sample section plane (out-of-plane tilt). When reading the pole figures, note that the sample plane also corresponds to the equator in the pole figure.

(ii) The relative volume of each orientation population (population volume) determined as the volume fraction *V*_f_ of crystallites close to a given fibre texture.

(iii) The texture index *J*_ODF_.

(iv) The *c*-axis orientation difference between different populations if more than one population is present.

**Figure 7:**
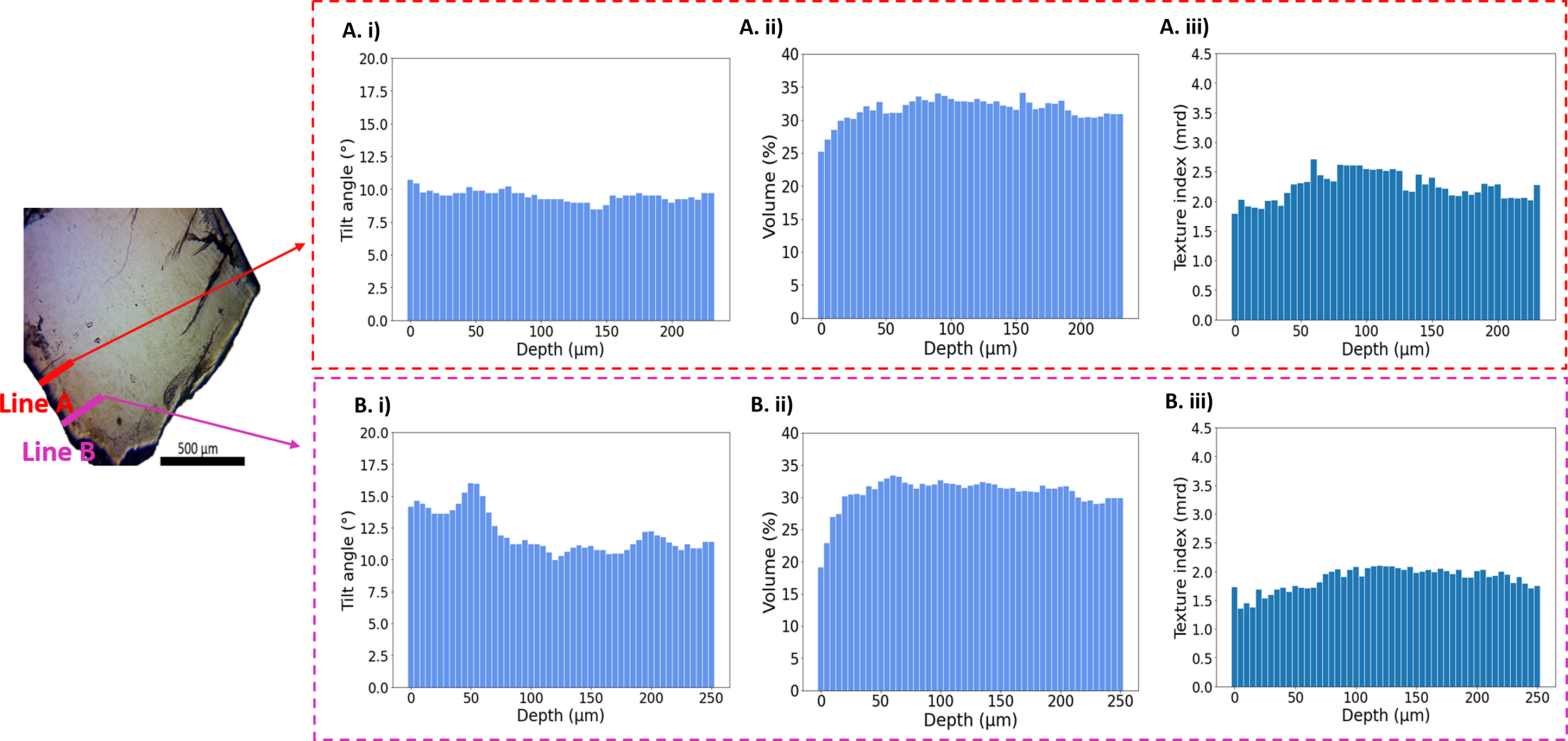
(Left) An optical microscope image of the demineralized tooth without peptide treatment (DC), indicating the positions of both line scans. (*A*)(i), (*A*)(ii) and (*A*)(iii) Histograms displaying the values of the tilt angle of the crystallites, the volume of crystallites having one fibre texture and the texture index, respectively, for Line A. (*B*)(i), (*B*)(ii) and (*B*)(iii) Histograms showing the values of the tilt angle of the crystallites, the volume of crystallites having one fibre texture and the texture index, respectively, for Line B.

In the artificially demineralized sample (DC), two lines spanning 230 and 250 µm in length and orientated perpendicularly to the enamel surface were scanned (left-hand side of Fig. 7 ▸). Notably, our analysis revealed a single crystallite orientation population at each point. The average *c*-axis orientation with respect to the sample plane (out-of-plane tilt) was approximately 9.5° in Line A and 12° in Line B [Figs. 7 ▸(*A*)(i) and 7 ▸(*B*)(i)]. Furthermore, when we assessed the population volume *V*_f_ [Figs. 7 ▸(*A*)(ii) and 7 ▸(*B*)(ii)] and texture index *J*_ODF_ [Figs. 7 ▸(*A*)(iii) and 7 ▸(*B*)(iii)], we observed a consistent decrease in both parameters within the initial 100 µm from the enamel surface compared with the adjacent enamel region. This observation suggests a disrupted alignment of crystallites within the demineralized zone.

**Figure 8:**
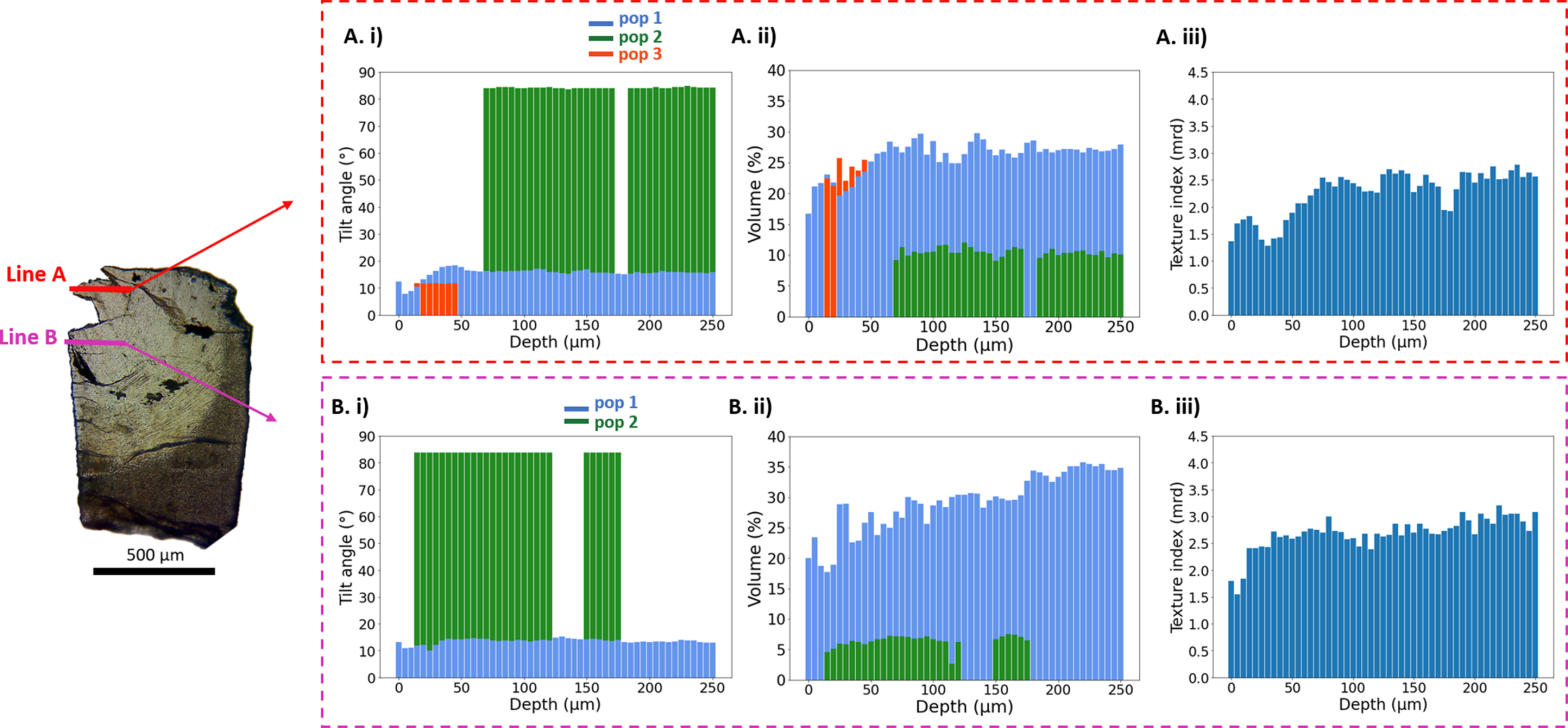
(Left) An optical microscope image of the STN-treated then demineralized sample (DS), highlighting both scanned lines. (*A*)(i), (*A*)(ii) and (*A*)(iii) Histograms showing the values of the tilt angle of the crystallites of different populations, the volume of crystallites having one fibre texture and the texture index, respectively, for Line A. (*B*)(i), (*B*)(ii) and (*B*)(iii) Histograms showing the values of the tilt angle of the crystallites, the volume of crystallites having one fibre texture and the texture index, respectively, for Line B.

In the STN-treated and then demineralized sample (DS), two lines measuring 250 µm each and oriented perpendicular to the enamel surface were scanned. Our analysis of PFs and ODFs revealed the presence of three distinct crystallite orientation populations. In Line A, a first orientation population (Pop1), with an orientation similar to that observed in sample DC, was consistently present throughout the scanned line with an average out-of-plane tilt angle of 16° [Fig. 8 ▸(*A*)(i)]. Additionally, we identified a second crystallite population (Pop2) located approximately 70 µm away from the enamel surface, with an average out-of-plane tilt of 94°. Pop2 was absent between the 70 and 80 µm marks. A third population of crystallites, Pop3, was present between 15 and 45 µm from the enamel surface, with an out-of-plane tilt angle of 12° on average. While the out-of-plane tilts of Pop1 and Pop 3 were similar, these populations showed a *c*-axis orientation difference of 36° on average. Detailed values are shown in Table 1 ▸.

In our analysis of the second line scan, Line B, we identified two populations similar to Pop1 and Pop 2 in Line A. While Pop1 was present in the whole scan line throughout the enamel, Pop2 was only present in the region 15–120 µm from the enamel surface and reappeared at a depth of 150–175 µm. Values of tilt angles of both populations throughout the enamel are shown in Fig. 8 ▸(*B*)(i) and Table 1 ▸.

Evaluation of the population volume *V*_f_ showed that Pop1 and Pop2 were the most prominent populations, with Pop2 being less prominent [Figs. 8 ▸(*A*)(ii) and 8 ▸(*B*)(ii)]. Further analysis showed a reduced texture up to a depth of 80 µm from the enamel surface in Line A and a similar decrease in texture in the first 15 µm from the surface in Line B, suggesting potential lower crystallite organization in these regions.

**Figure 9:**
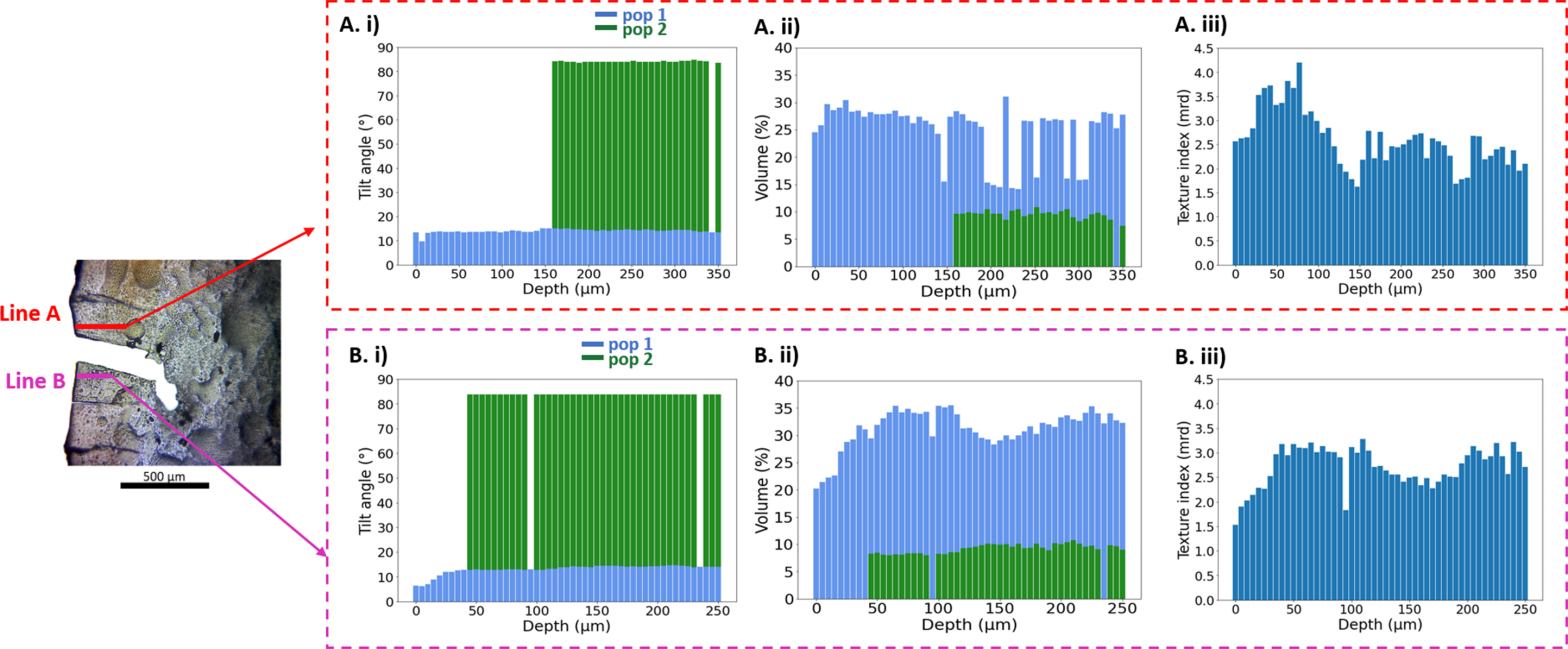
(Left) An optical microscope image of the HTN-treated then demineralized tooth (DH), with both scanned lines indicated. (*A*)(i), (*A*)(ii) and (*A*)(iii) Histograms showing the values of the tilt angle of the crystallites, the volume of crystallites having one fibre texture and the texture index, respectively, for Line A. (*B*)(i), (*B*)(ii) and (*B*)(iii) Histograms showing the values of the tilt angle of the crystallites, the volume of crystallites having one fibre texture and the texture index, respectively, for Line B.

In the demineralized and then HTN-treated tooth (DH), again two lines were scanned. Line A measures 350 µm from the enamel surface while Line B was probed to a depth of 250 µm from the enamel surface. In both of these lines, the analysis of PFs and ODFs revealed the presence of two distinct crystallite populations. The out-of-plane tilt angle values of Pop1 and Pop2 throughout Line A and Line B are displayed in Figs. 9 ▸(*A*)(i) and 9 ▸(*B*)(i), respectively. Notably, in Line A, Pop2 became evident at a depth of 160 µm from the enamel surface, whereas in Line B, Pop2 started to manifest at a depth of 45 µm from the enamel surface. Additionally, we assessed the angle of *c*-axis orientation difference between Pop1 and Pop2 across the enamel (see values in Table 1 ▸). In Figs. 9 ▸(*A*)(ii) and 9 ▸(*B*)(ii), the relative volume fractions of the two populations are shown. Again, Pop1 was found to be more prominent than Pop2. Our texture index analysis of Line B revealed reduced values within the first 30 µm from the enamel surface, followed by another decrease in texture index between 100 and 160 µm depth. These findings suggest significant modifications in crystallite alignment along Line A. Similarly in Line B, our texture analysis revealed a lower texture index up to a depth of 50 µm from the enamel surface, with another reduction in texture index observed between the 125 and 175 µm depths, implying potential alterations in crystallite organization along Line B as well.

In Table 1 ▸, the mean orientation parameters of the different crystallite populations in each sample are presented. Standard deviations of the mean value over all positions sampled are given as ±.

## 4. Discussion

In this study, we have aimed to demonstrate the potential of the energy-dispersive Laue diffraction technique as a powerful tool for investigating complex textures found in biomineralized tissues such as dental enamel. By utilizing this technique, we can overcome the limitations of traditional diffraction methods and obtain pole figures with a notable coverage of reciprocal space directly and in one shot without any sample rotation (Grünewald *et al.*, 2016 ▸). In this way we were able to scan enamel samples with high spatial resolution and reveal texture inhomogeneities on the micrometre scale. We could show that different fibre texture populations were present along the scan lines and that their orientations and combinations varied within a few micrometres. Such variations would be challenging to resolve if sample rotation had to be employed, due to the effect of rotation smearing.

Furthermore, there is a clear gain in information when comparing our approach with conventional monochromatic 2D X-ray diffraction scanning approaches. Results from 2D XRD, if printed onto a pole figure, would yield a single line only, corresponding to the used energy *E* (and to the scattering angle 2θ for a given reflection). Any reflection spots not falling on this line would not be visible. This makes it more difficult to detect out-of-plane tilts of crystallites, which are readily seen and quantitatively analysed in our approach. As shown in Fig. 5 ▸, the 3D crystallographic orientation of different fibre populations can be directly evaluated from the experimental data in every pixel of the 2D scan.

Nevertheless, useful texture information has also been extracted in the past by XRD using area detectors by considering the azimuthal intensity distributions and collecting a large number of reflections. Rietveld refinement is often used in the literature and is also of great value to gain texture information in dental enamel, as shown for example by Al-Jawad *et al.* (2007 ▸).

Other approaches to detect out-of-plane tilts of crystallites were based on geometric considerations taking into account the curvature of the Ewald sphere, such as, for example, to obtain information about the tilt angle of cellulose fibres in the wood cell wall (Lichtenegger *et al.*, 1999 ▸) by inferring a fibre texture. More recently, a related approach was used in combination with modelling to analyse the orientation of chitin fibrils in mantis shrimp (Zhang *et al.*, 2017 ▸, 2016 ▸). Another method based on computational extraction of tilt data from 2D diffraction patterns was presented by Méheust *et al.* (2006 ▸). This method assumes that the ODF of the crystallites depends solely on the deviation of crystallite orientation from a fixed reference direction.

In general, in all 2D XRD experiments without rotation, the reflections in question have to fall on the line in the pole figure accessible by monochromatic XRD, while EDLD offers a broader area of coverage in reciprocal space. Since this coverage is available for different reflections oriented at different angles with respect to each other, this provides much richer information for the calculation of the ODF and therefore the reconstruction of full pole figures. This is of particular advantage in cases of complex textures with several main orientations present, as in our enamel samples. In this way, we can obtain quantitative texture information such as the texture index and the relative volume fraction of crystallites present in a certain orientation.

In EDLD, not only the orientation but also the type of texture (here fibre texture) is well represented in the ODF and reconstructed pole figures, even if the experimental coverage of reciprocal space is limited by the available energy and diffraction angles 2θ (see Fig. 4 ▸). This makes EDLD a powerful technique with the potential to identify and analyse completely unknown types of textures. It should be mentioned that the rather recently developed methods of small- or wide-angle tensor tomography can also shed light on the 3D orientation of crystallites in biological samples (Grünewald *et al.*, 2020 ▸; Liebi *et al.*, 2015 ▸; Schaff *et al.*, 2015 ▸). Nevertheless, these methods have been restricted to detecting a single orientation in one voxel so far, thus not providing pole figures or ODFs that would cover the complexity seen here.

Further improvements could be made by obtaining a larger coverage of reciprocal space, which would reduce reconstruction artefacts and increase the quality of the results. This could be achieved by employing a larger range of energies and scattering angles. In our case we were limited by the small size of the active detector area (1 × 1 cm) and by the available energy range. Table 2 ▸ displays the experimentally accessible range for our experiment. In the small-angle regime, it is limited by the maximum energy, in our case given by the energy cutoff of the setup of the Laue station on BM32. In a different setup, higher energies can be achieved, in which case the quantum efficiency of the detector becomes the limiting factor (Ordavo *et al.*, 2011 ▸).

In the large-angle regime the accessible range is constrained by the detector size and the usable sample-to-detector distance. Such limitations can be overcome in the future by using larger sized energy-dispersive area detectors, as demonstrated, for example, by Strüder *et al.* (2010 ▸). Finally, using more reflections for estimation of the ODF and reconstructed PFs is always beneficial.

In this proof-of-concept study, we selected human dental enamel as our sample of interest. By employing EDLD, our goal was to gain a deeper understanding of the mechanisms by which two peptides, histatin and statherin, influence the demineralization of enamel. These peptides have been previously shown to slow down the demineralization process by binding to hy­droxy­apatite crystallites (Kosoric *et al.*, 2007 ▸; Shah *et al.*, 2011 ▸; Siqueira *et al.*, 2010 ▸), but the exact mechanisms underlying their protective effects are not fully understood.

Our study revealed notable differences in the demineralization effects on the crystallographic structure of enamel when treated with and without the two peptides. The overall texture index of the demineralized enamel sample without salivary proteins was lower than for those treated with HTN or STN (Table 1 ▸). In particular, the texture index was lower near the enamel surface, suggesting alterations in crystallite organization in this region (Figs. 7 ▸–9 ▸ ▸).

This finding aligns with a previous study on human enamel using 2D synchrotron XRD with a beam diameter of 20 µm. That study reported a significant reduction in crystallographic texture throughout the depth of enamel following demineralization with 1% acetic acid solution (pH 4, 100 ml) (Siddiqui *et al.*, 2014 ▸). Conversely, another study employing small- and wide-angle X-ray scattering techniques, with a beam size of 150 µm, noted an increase in crystal alignment following demineralization of human dental enamel using 10% lactic acid solution (pH 2.2, 100 µl) (Sui *et al.*, 2018 ▸). These conflicting findings regarding the behaviour of enamel crystallites in response to demineralization could potentially be attributed to variations in composition, pH and concentration of the employed demineralization solutions.

**Figure 10:**
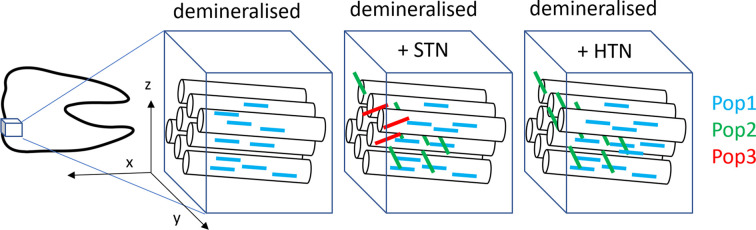
The orientation of crystallite populations in enamel. (Left) A sketch of a tooth with an indication of the measured sample volume. The tooth was sectioned in the *xy* plane and the incident beam hit the sample in the −**z** direction. Enamel prisms are assumed to be oriented perpendicular to the enamel surface, according to results from the literature. The measured 3D orientation of different crystallite populations is indicated by coloured glyphs: Pop1 roughly in the **x** direction with some tilt, Pop3 roughly in the **y** direction and Pop2 in an oblique direction.

Given the capabilities of EDLD, we were able to determine the 3D orientation of crystallite populations present in each individual sample in a position-resolved way. In all samples a main orientation population (Pop1, blue glyphs in Fig. 10 ▸) roughly perpendicular to the enamel surface was observed, which corresponds to the longitudinal direction of enamel prisms, as described in the literature (Al-Jawad *et al.*, 2007 ▸). However, the STN-treated sample showed two additional orientation populations (Pop2 and Pop3), with Pop3 (oblique orientation, red glyphs in Fig. 10 ▸) occurring very close to the enamel surface and only in one scan line. Pop2 corresponds to an orientation along the enamel surface and perpendicular to the presumed rod direction (green glyphs in Fig. 10 ▸). It was present in a major part of the sample treated with STN. Similarly, the HTN-treated sample contained Pop2 in addition to Pop1. Structural complexity and the presence of different orientation populations have also been reported in healthy enamel (Al-Mosawi *et al.*, 2018 ▸). Although a general interpretation is difficult with only one sample for each treatment, it is notable that reduced complexity with only one population (Pop1) was observed both in the demineralized sample with lower texture index and in the first 20–150 µm of the STN- and HTN-treated samples (Figs. 8 ▸ and 9 ▸), where the texture index was also lower.

In the literature, it has been found that enamel demineralization by acid treatment can give rise to different dissolution patterns: Type 1 shows loss in prism cores (also referred to as prism heads), Type 2 displays mineral loss at the prism periphery (also referred to as prism tails or inter-prismatic regions) with intact prism cores, and Type 3 exhibits random demineralization patterns (Harper *et al.*, 2021 ▸; Silverstone *et al.*, 1975 ▸; Wang *et al.*, 2005 ▸). These patterns were found to be influenced by crystal orientation and varied depending on the demineralization solutions used (Silverstone *et al.*, 1975 ▸). Demineralization has been reported to be initially Type 2, while Type 1 becomes more dominant as lesions progress (Pearce & Nelson, 1989 ▸). While the crystallite *c*-axis orientation in the prism core has been demonstrated to follow predominantly the prism direction (here Pop1), the inter-prismatic regions show crystallites with deviating directions (White *et al.*, 2001 ▸; Beniash *et al.*, 2019 ▸). A predominant dissolution of the inter-prismatic regions would reduce the local complexity, as observed in the demineralized sample and the surface layers of each sample. The presence of the other populations in the STN- and HTN-treated samples points towards a protective effect of STN and HTN specifically in those regions. It has been established that, compared with prism cores, inter-prismatic regions tend to have increased inter-crystalline space (Shore *et al.*, 1995*b* ▸) and higher organic content (Ang *et al.*, 2012 ▸). The notion that inter-prismatic enamel may possess higher organic content suggests that the initial demineralization process, particularly Type 2 patterns involving mineral loss in the inter-prismatic region, could be influenced by this organic composition. In this context, the presence of HTN and STN may play a protective role by preserving or replacing the organic component. The observed presence of Pop1 only in the surface layers is also compatible with a more progressed dissolution of the inter-prismatic regions close to the enamel surface where the acid attack starts. These findings align with previous literature reports that highlight the roles of STN (Kosoric *et al.*, 2007 ▸; Shah *et al.*, 2011 ▸) and HTN (Siqueira *et al.*, 2010 ▸) in preventing enamel demineralization.

## 5. Conclusion

We have demonstrated the potential of EDLD for quantitative spatially resolved texture analysis in complex biological materials, allowing 2D mapping of the texture without the need for sample rotation. The outcomes of our study serve as a proof of concept for the application of EDLD for studying the demineralization process of dental enamel. The results obtained shed new light on the effects of HTN and STN on the crystallographic structure of enamel. Further research utilizing EDLD and complementary techniques can deepen our understanding of enamel structure and contribute to the development of preventative strategies for dental health.

## Supplementary Material

Additional figures and notes. DOI: 10.1107/S1600576724007180/xx5050sup1.pdf

## Figures and Tables

**Table 1:** Mean values of the out-of-plane tilt of Pop1, Pop2 and Pop3, misorientation angles between Pop1–Pop2 and Pop1–Pop3, and texture index obtained by averaging lines A and B throughout the enamel

	DC	DS	DH
Out-of-plane tilt, Pop1 (°)	10.9 ± 0.35	14.60 ± 1.43	13.50 ± 1.35
Out-of-plane tilt, Pop2 (°)		84.00 ± 0.10	83.90 ± 0.17
Out-of-plane tilt, Pop3 (°)		11.70 ± 0.06	
Relative orientation (Pop1–Pop2) (°)		70.0 ± 1.95	71.17 ± 2.34
Relative orientation (Pop1–Pop3) (°)		36.0 ± 3.80	
Texture index (multiples of random distribution, mrd)	2.00 ± 0.21	2.32 ± 0.20	2.90 ± 0.46

**Table 2:** Upper and lower limits of coverage in pole figures Note that θ_max_ is constant and given by the maximum accessible scattering angle 2θ. *E*_max_ is also constant due to the energy cutoff of the KB mirror.

		Upper limit in PF		Lower limit in PF	
Reflection	*d* spacing (Å)	*E*_min_ (keV)	θ_max_ (°)	*E*_max_ (keV)	θ_min_ (°)
002	3.4432	5.2	20	23	4.49
211	2.8135	6.4	20	23	5.50
310	2.2624	8.0	20	23	6.84

## References

[bb1] Abboud, A., AlHassan, A., Dönges, B., Micha, J. S., Hartmann, R., Strüder, L., Christ, H.-J. & Pietsch, U. (2021). *Int. J. Fatigue*, **151**, 106358.

[bb2] Abboud, A., Kirchlechner, C., Keckes, J., Conka Nurdan, T., Send, S., Micha, J. S., Ulrich, O., Hartmann, R., Strüder, L. & Pietsch, U. (2017). *J. Appl. Cryst.* **50**, 901–908. 10.1107/S1600576717005581 PMC5458596 28656042

[bb3] Abboud, A., Kirchlechner, C., Send, S., Micha, J. S., Ulrich, O., Pashniak, N., Strüder, L., Keckes, J. & Pietsch, U. (2014). *Rev. Sci. Instrum.* **85**, 113901. 10.1063/1.4900482 25430118

[bb4] Al-Jawad, M., Steuwer, A., Kilcoyne, S. H., Shore, R. C., Cywinski, R. & Wood, D. J. (2007). *Biomaterials*, **28**, 2908–2914. 10.1016/j.biomaterials.2007.02.019 17367851

[bb5] Almeida, P. D. V. de, Grégio, A. M. T., Machado, M., De Lima, A. A. S. & Azevedo, L. R. (2008). *J. Contemp. Dent. Pract.* **9**, 72–80. 18335122

[bb6] Almer, J. D. & Stock, S. R. (2005). *J. Struct. Biol.* **152**, 14–27. 10.1016/j.jsb.2005.08.003 16183302

[bb7] Al-Mosawi, M., Davis, G. R., Bushby, A., Montgomery, J., Beaumont, J. & Al-Jawad, M. (2018). *Sci. Rep.* **8**, 14449. 10.1038/s41598-018-32425-y PMC6160435 30262903

[bb8] Anderson, P., Hector, M. P. & Rampersad, M. A. (2001). *Int. J. Paed. Dent.* **11**, 266–273. 10.1046/j.1365-263x.2001.00293.x 11570442

[bb9] Ang, S. F., Saadatmand, M., Swain, M. V., Klocke, A. & Schneider, G. A. (2012). *J. Mater. Res.* **27**, 448–456.

[bb10] Beniash, E., Stifler, C. A., Sun, C.-Y., Jung, G. S., Qin, Z., Buehler, M. J. & Gilbert, P. U. P. A. (2019). *Nat. Commun.* **10**, 4383. 10.1038/s41467-019-12185-7 PMC6763454 31558712

[bb11] Besnard, C., Marie, A., Sasidharan, S., Harper, R. A., Shelton, R. M., Landini, G. & Korsunsky, A. M. (2023). *Dent. J.* **11**, 98. 10.3390/dj11040098 PMC10137518 37185477

[bb12] Boyde, A. (1964). PhD thesis, University of London, UK.

[bb13] Boyde, A. (1997). *Ciba Found. Symp.* **205**, 18–31. 10.1002/9780470515303.ch3 9189615

[bb14] Chen, P.-H., Tseng, Y.-H., Mou, Y., Tsai, Y.-L., Guo, S.-M., Huang, S.-J., Yu, S. S.-F. & Chan, J. C. (2008). *J. Am. Chem. Soc.* **130**, 2862–2868. 10.1021/ja076607y 18266360

[bb15] Cho, J.-H., Rollett, A. D. & Oh, K. H. (2004). *Metall. Mater. Trans. A*, **35**, 1075–1086.

[bb16] Cortie, M. B. (1997). *Textures Microstruct.* **29**, 698480.

[bb17] Cullity, B. D. (1978). *Elements of X-ray Diffraction*, 2nd ed. Reading: Addison–Wesley.

[bb18] Dodds, M. W., Johnson, D. A. & Yeh, C.-K. (2005). *J. Dent.* **33**, 223–233. 10.1016/j.jdent.2004.10.009 15725522

[bb19] Dowd, F. J. (1999). *Dent. Clin. North Am.* **43**, 579–597. 10553245

[bb20] Elliott, J. C. (1997). *Ciba Found. Symp.* **205**, 54–72. 9189617

[bb21] Elliott, J. C., Wong, F. S., Anderson, P., Davis, G. R. & Dowker, S. E. (1998). *Connect. Tissue Res.* **38**, 61–72. 10.3109/03008209809017022 11063016

[bb22] Free, R., DeRocher, K., Cooley, V., Xu, R., Stock, S. R. & Joester, D. (2022). *Proc. Natl Acad. Sci. USA*, **119**, e2211285119. 10.1073/pnas.2211285119 PMC9907129 36534796

[bb24] Grünewald, T. A., Liebi, M., Wittig, N. K., Johannes, A., Sikjaer, T., Rejnmark, L., Gao, Z., Rosenthal, M., Guizar-Sicairos, M., Birkedal, H. & Burghammer, M. (2020). *Sci. Adv.* **6**, eaba4171. 10.1126/sciadv.aba4171 PMC7292642 32582855

[bb23] Grünewald, T. A., Rennhofer, H., Tack, P., Garrevoet, J., Wermeille, D., Thompson, P., Bras, W., Vincze, L. & Lichtenegger, H. C. (2016). *Angew. Chem. Int. Ed.* **55**, 12190–12194. 10.1002/anie.201603784 27483396

[bb25] Habelitz, S., Marshall, S. J., Marshall, G. W. & Balooch, M. (2001). *Arch. Oral Biol.* **46**, 173–183. 10.1016/s0003-9969(00)00089-3 11163325

[bb26] Harper, R. A., Shelton, R. M., James, J. D., Salvati, E., Besnard, C., Korsunsky, A. M. & Landini, G. (2021). *Acta Biomaterialia*, **120**, 240–248. 10.1016/j.actbio.2020.04.045 32438107

[bb27] Hielscher, R. & Schaeben, H. (2008). *J. Appl. Cryst.* **41**, 1024–1037.

[bb28] Ionescu, A. C., Degli Esposti, L., Iafisco, M. & Brambilla, E. (2022). *Sci. Rep.* **12**, 5994. 10.1038/s41598-022-09787-5 PMC8994765 35397624

[bb29] Jaschouz, D., Paris, O., Roschger, P., Hwang, H.-S. & Fratzl, P. (2003). *J. Appl. Cryst.* **36**, 494–498.

[bb30] Kaufman, E. & Lamster, I. B. (2002). *Crit. Rev. Oral Biol. Med.* **13**, 197–212. 10.1177/154411130201300209 12097361

[bb31] Kerebel, B., Daculsi, G. & Kerebel, L. (1979). *J. Dent. Res.* **58**, 844–851. 10.1177/00220345790580023701 283126

[bb32] Kieffer, J., Valls, V., Blanc, N. & Hennig, C. (2020). *J. Synchrotron Rad.* **27**, 558–566. 10.1107/S1600577520000776 PMC7842211 32153298

[bb33] Koblischka-Veneva, A., Koblischka, M. R., Schmauch, J. & Hannig, M. (2019). *IOP Conf. Ser. Mater. Sci. Eng.* **625**, 012006.

[bb34] Koenigswald, W. von, Rensberger, J. M. & Pretzschner, H. U. (1987). *Nature*, **328**, 150–152. 10.1038/328150a0 3600790

[bb35] Kosoric, J., Williams, R. A. D., Hector, M. P. & Anderson, P. (2007). *Int. J. Pept. Res. Ther.* **13**, 497–503.

[bb37] Lenander-Lumikari, M. & Loimaranta, V. (2000). *Adv. Dent. Res.* **14**, 40–47. 10.1177/08959374000140010601 11842922

[bb38] Lichtenegger, H., Müller, M., Paris, O., Riekel, C. & Fratzl, P. (1999). *J. Appl. Cryst.* **32**, 1127–1133.

[bb39] Liebi, M., Georgiadis, M., Menzel, A., Schneider, P., Kohlbrecher, J., Bunk, O. & Guizar-Sicairos, M. (2015). *Nature*, **527**, 349–352. 10.1038/nature16056 26581291

[bb40] Machiulskiene, V., Campus, G., Carvalho, J. C., Dige, I., Ekstrand, K. R., Jablonski-Momeni, A., Maltz, M., Manton, D. J., Martignon, S., Martinez-Mier, E. A., Pitts, N. B., Schulte, A. G., Splieth, C. H., Tenuta, L. M. A., Ferreira Zandona, A. & Nyvad, B. (2020). *Caries Res.* **54**, 7–14. 10.1159/000503309 31590168

[bb41] Mainprice, D., Bachmann, F., Hielscher, R. & Schaeben, H. (2015). *Geol. Soc. London Spec. Publ.* **409**, 251–271.

[bb42] Makrodimitris, K., Masica, D. L., Kim, E. T. & Gray, J. J. (2007). *J. Am. Chem. Soc.* **129**, 13713–13722. 10.1021/ja074602v 17929924

[bb43] Mann, S. (2001). *Biomineralization – Principles and Concepts in Bioinorganic Materials Chemistry.* Oxford University Press.

[bb44] Margolis, H. & Moreno, E. (1992). *J. Dent. Res.* **71**, 1776–1784. 10.1177/00220345920710110301 1401439

[bb45] Méheust, Y., Knudsen, K. D. & Fossum, J. O. (2006). *J. Appl. Cryst.* **39**, 661–670.

[bb46] Ordavo, I., Ihle, S., Arkadiev, V., Scharf, O., Soltau, H., Bjeoumikhov, A., Bjeoumikhova, S., Buzanich, G., Gubzhokov, R., Günther, A., Hartmann, R., Holl, P., Kimmel, N., Kühbacher, M., Lang, M., Langhoff, N., Liebel, A., Radtke, M., Reinholz, U., Riesemeier, H., Schaller, G., Schopper, F., Strüder, L., Thamm, C. & Wedell, R. (2011). *Nucl. Instrum. Methods Phys. Res. A*, **654**, 250–257.

[bb47] Park, J. S., Chen, H., James, K. C., Natanson, L. J. & Stock, S. R. (2022). *J. Mech. Behav. Biomed. Mater.* **136**, 105506. 10.1016/j.jmbbm.2022.105506 36228402

[bb48] Pearce, E. I. F. & Nelson, D. G. A. (1989). *J. Dent. Res.* **68**, 113–118. 10.1177/00220345890680020301 2918131

[bb49] Poole, D. & Brooks, A. (1961). *Arch. Oral Biol.* **5**, 14–26. 10.1016/0003-9969(61)90110-8 13972177

[bb50] Raghunathan, V., Gibson, J. M., Goobes, G., Popham, J. M., Louie, E. A., Stayton, P. S. & Drobny, G. P. (2006). *J. Phys. Chem. B*, **110**, 9324–9332. 10.1021/jp056644g 16671751

[bb51] Reznikov, N., Bilton, M., Lari, L., Stevens, M. M. & Kröger, R. (2018). *Science*, **360**, eaao2189. 10.1126/science.aao2189 PMC6037297 29724924

[bb52] Robinson, C., Kirkham, J., Brookes, S. J. & Shore, R. C. (1995). *Dental Enamel: Formation to Destruction*, edited by C. Robinson, J. Kirkham & R. C. Shore, pp. 167–191. Boca Raton: CRC Press.

[bb54] Schaff, F., Bech, M., Zaslansky, P., Jud, C., Liebi, M., Guizar-Sicairos, M. & Pfeiffer, F. (2015). *Nature*, **527**, 353–356. 10.1038/nature16060 26581292

[bb55] Send, S., Abboud, A., Leitenberger, W., Weiss, M. S., Hartmann, R., Strüder, L. & Pietsch, U. (2012). *J. Appl. Cryst.* **45**, 517–522.

[bb56] Shah, S., Kosoric, J., Hector, M. P. & Anderson, P. (2011). *Eur. J. Oral Sci.* **119**(Suppl. 1), 13–18. 10.1111/j.1600-0722.2011.00899.x 22243221

[bb57] Shore, R. C., Robinson, C., Kirkham, J. & Brookes, S. J. (1995*a*). *Dental Enamel: Formation to Destruction*, edited by C. Robinson, J. Kirkham & R. C. Shore, pp. 135–150. Boca Raton: CRC Press.

[bb58] Shore, R. C., Robinson, C., Kirkham, J. & Brookes, S. J. (1995*b*). *Dental Enamel: Formation to Destruction*, edited by C. Robinson, J. Kirkham & R. C. Shore, pp. 151–166. Boca Raton: CRC Press.

[bb59] Siddiqui, S., Anderson, P. & Al-Jawad, M. (2014). *PLoS One*, **9**, e108879. 10.1371/journal.pone.0108879 PMC4215832 25360532

[bb60] Silverstone, L. M., Saxton, C. A., Dogon, I. L. & Fejerskov, O. (1975). *Caries Res.* **9**, 373–387. 10.1159/000260179 1055640

[bb61] Simmer, J. P., Richardson, A. S., Hu, Y.-Y., Smith, C. E. & Ching-Chun Hu, J. (2012). *Int. J. Oral Sci.* **4**, 129–134. 10.1038/ijos.2012.59 PMC3464985 22996272

[bb62] Siqueira, W. L., Margolis, H. C., Helmerhorst, E. J., Mendes, F. M. & Oppenheim, F. G. (2010). *J. Dent. Res.* **89**, 626–630. 10.1177/0022034510363384 PMC2873135 20351356

[bb63] Stock, S. R., Morse, P. E., Stock, M. K., James, K. C., Natanson, L. J., Chen, H., Shevchenko, P. D., Maxey, E. R., Antipova, O. A. & Park, J. S. (2022). *J. Med. Imag.* **9**, 031504. 10.1117/1.JMI.9.3.031504 PMC8809398 35127969

[bb64] Strüder, L., Epp, S., Rolles, D., Hartmann, R., Holl, P., Lutz, G., Soltau, H., Eckart, R., Reich, C., Heinzinger, K., Thamm, C., Rudenko, A., Krasniqi, F., Kühnel, K.-U., Bauer, C., Schröter, C.-D., Moshammer, R., Techert, S., Miessner, D., Porro, M., Hälker, O., Meidinger, N., Kimmel, N., Andritschke, R., Schopper, F., Weidenspointner, G., Ziegler, A., Pietschner, D., Herrmann, S., Pietsch, U., Walenta, A., Leitenberger, W., Bostedt, C., Möller, T., Rupp, D., Adolph, M., Graafsma, H., Hirsemann, H., Gärtner, K., Richter, R., Foucar, L., Shoeman, R. L., Schlichting, I. & Ullrich, J. (2010). *Nucl. Instrum. Methods Phys. Res. A*, **614**, 483–496.

[bb65] Sui, T., Salvati, E., Harper, R. A., Zhang, H., Shelton, R. M., Landini, G. & Korsunsky, A. M. (2018). *Acta Biomaterialia*, **77**, 333–341. 10.1016/j.actbio.2018.07.027 30026103

[bb66] Tamura, N. & Gilbert, P. U. P. A. (2013). *Methods Enzymol.* **532**, 501–531. 10.1016/B978-0-12-416617-2.00021-7 24188780

[bb67] Wagermaier, W., Gupta, H. S., Gourrier, A., Paris, O., Roschger, P., Burghammer, M., Riekel, C. & Fratzl, P. (2007). *J. Appl. Cryst.* **40**, 115–120.

[bb68] Wang, L., Tang, R., Bonstein, T., Orme, C. A., Bush, P. J. & Nancollas, G. H. (2005). *J. Phys. Chem. B*, **109**, 999–1005. 10.1021/jp046451d 16866472

[bb69] Weiner, S. (2008). *J. Struct. Biol.* **163**, 229–234. 10.1016/j.jsb.2008.02.001 18359639

[bb70] Wenk, H. R. & Heidelbach, F. (1999). *Bone*, **24**, 361–369. 10.1016/s8756-3282(98)00192-6 10221548

[bb71] White, S. N., Luo, W., Paine, M. L., Fong, H., Sarikaya, M. & Snead, M. L. (2001). *J. Dent. Res.* **80**, 321–326. 10.1177/00220345010800010501 11269723

[bb72] Zhang, Y., De Falco, P., Wang, Y., Barbieri, E., Paris, O., Terrill, N. J., Falkenberg, G., Pugno, N. M. & Gupta, H. S. (2017). *Nanoscale*, **9**, 11249–11260. 10.1039/c7nr02139a 28753215

[bb73] Zhang, Y., Paris, O., Terrill, N. J. & Gupta, H. S. (2016). *Sci. Rep.* **6**, 26249. 10.1038/srep26249 PMC4876318 27211574

