# Supplementary material for: Energy-dispersive Laue diffraction analysis of the influence of statherin and histatin on the crystallographic texture during human dental enamel demineralization
Source: J Appl Crystallogr. 2024 Sep 25;57(Pt 5):1514–27. doi: 10.1107/S1600576724007180 (PMC11460385; doi:10.1107/S1600576724007180)
Supplement: Supplementary file 1 [file j-57-01514-sup1.pdf]

JOURNAL OF
APPLIED
CRYSTALLOGRAPHY

Volume 57 (2024)

Supporting information for article:

Energy-dispersive Laue diffraction analysis of the influence of statherin and histatin on the crystallographic texture during human dental enamel demineralization

C. Sakr, M. Al-Mosawi, T. Gruenewald, P. Cook, P. Tack, L. Vincze, J.-S. Micha, P. Anderson, M. Al-Jawad and H. C. Lichtenegger

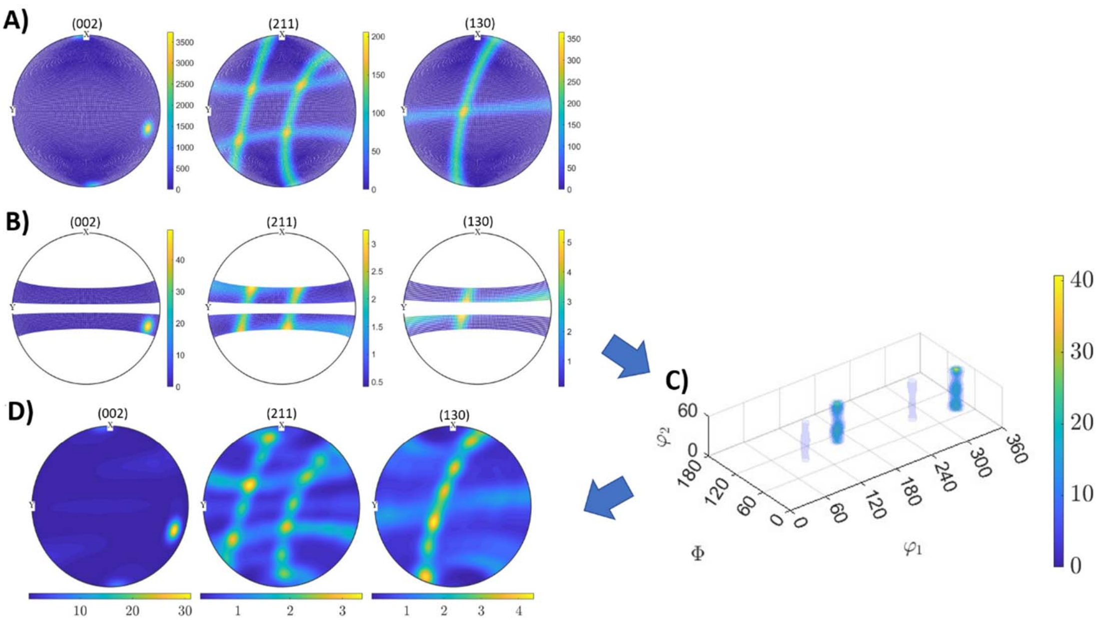

Figure S1 A) Simulated full PFs, B) incomplete PFs in the experimentally accessible range and C) estimated ODF from incomplete PFs, D) reconstructed pole figures from ODF obtained from partial pole figures.

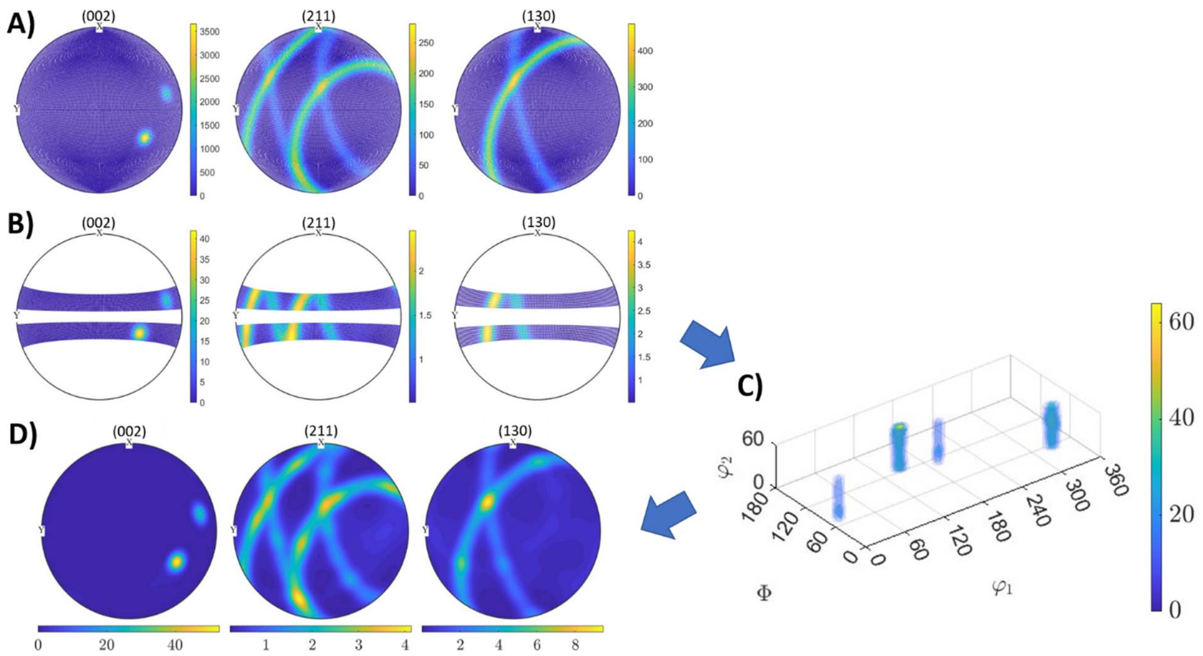

Figure S2 A) Simulated full PFs, B) incomplete PFs in the experimentally accessible range and C) estimated ODF from incomplete PFs, D) reconstructed pole figures from ODF obtained from partial pole figures.

Note S1

The specimens were embedded in fast curing acrylic cold mounting resin (ClaroCit Kit, Struers, Ballerup, Denmark). Each of the embedded teeth was cut through the mid-point perpendicular to the bucco-lingual surface using a Struers Accutom-5 diamond saw (Struers, Ballerup, Denmark) to produce a 300 µm thick mid slice for each tooth. Slices were then wet-polished down to 20 µm using 800 grit silicon carbide abrasive paper with an adhesive backing (Wetordry Tri-M-Ite paper, 3M, MN, USA). Briefly, a silicon carbide abrasive paper was affixed to a benchtop. Another piece of abrasive paper was cut into a rectangular shape and glued to a 25 x 75 mm microscope slide (Corning, NY, USA). Distilled water was added to the abrasive paper on the benchtop, and the tooth sample was placed on this surface. The microscope slide with the attached abrasive paper

was used to sandwich the tooth between the two pieces of abrasive paper. The slide was moved slowly in a circular motion by hand to wet-polish the tooth. The thickness of the tooth was periodically measured using a ball micrometer (Mitutoyo, Kawasaki, Japan) until the desired thickness of 20 µm was achieved. Due to the minimal thickness, the enamel often dissociated from the dentine, occasionally resulting in small isolated pieces of enamel. The samples were then ultrasonically cleaned in distilled water for 15 minutes using an ultrasonic bath (Kerry PUL-125, Guyson International Ltd., North Yorkshire, UK) to remove surface debris.

Note S2

The data were normalised to account for variations in incident intensity, detector sensitivity and sample absorption at different energies and background were corrected before further evaluation. Accurate calculation of the attenuation is a challenge, in particular in biological samples. Furthermore, knowing the spectrum of the incident beam is essential to normalize the data. Given the intensity of a white synchrotron beam, no direct measurement of the incident spectrum was possible. Therefore, a phenomenological experimental approach was used, considering the diffuse scatter signal present in each diffraction pattern and assuming it to be representative of the incident beam spectrum, already attenuated by the sample. In this way, neither the exact incident spectrum, nor the attenuation have to be determined separately. Moreover, the experimentally determined diffuse scatter signal already reflects the detector efficiency, which depends on the energy and which would otherwise also have to be determined separately.

Due to the high resolution given by the pixelated detector, identifying q ranges where no diffraction signal occurred is possible. After the azimuthal integration of the diffraction data, a non diffraction q range was selected. The intensity was integrated over the selected q range and the full azimuth

and plotted versus the energy. In result a spectrum is obtained which contains fluorescence peaks superimposed to a smooth diffuse scatter spectrum. The raw data was then normalized by first subtracting the fluorescence signal and then by dividing the diffraction data at each energy with the intensity of the diffuse scatter.
